# Remaining Useful Life Prediction for Two-Phase Nonlinear Degrading Systems with Three-Source Variability

**DOI:** 10.3390/s24010165

**Published:** 2023-12-27

**Authors:** Xuemiao Cui, Jiping Lu, Yafeng Han

**Affiliations:** School of Mechanical Engineering, Beijing Institute of Technology, Beijing 100081, China; cuixuemiao@bit.edu.cn (X.C.); jipinglu@bit.edu.cn (J.L.)

**Keywords:** degradation modeling, nonlinear Wiener process, variability, uncertainty, remaining useful life, prognostics

## Abstract

Recently, the estimation of remaining useful life (RUL) for two-phase nonlinear degrading devices has shown rising momentum for ensuring their safe and reliable operation. The degradation processes of such systems are influenced by the temporal variability, unit-to-unit variability, and measurement variability jointly. However, current studies only consider these three sources of variability partially. To this end, this paper presents a two-phase nonlinear degradation model with three-source variability based on the nonlinear Wiener process. Then, the approximate analytical solution of the RUL with three-source variability is derived under the concept of the first passage time (FPT). For better implementation, the offline model parameter estimation is conducted by the maximum likelihood estimation (MLE), and the Bayesian rule in conjunction with the Kalman filtering (KF) algorithm are utilized for the online model updating. Finally, the effectiveness of the proposed approach is validated through a numerical example and a practical case study of the capacitor degradation data. The results show that it is necessary to incorporate three-source variability simultaneously into the RUL prediction of the two-phase nonlinear degrading systems.

## 1. Introduction

With the rapid development of technology, the equipment in the fields of aerospace, high-speed rail, and ship manufacturing tend to be large-scale, complex, sophisticated, and long-life, which puts forward higher requirements for system reliability [1,2]. Recently, owing to the important role in realizing predictive maintenance, improving system reliability, reducing maintenance costs, and avoiding catastrophic eventualities, prognostics and health management (PHM) has obtained extensive attention both in academia and industry [3,4,5]. As an essential part of PHM, the remaining useful life (RUL) is defined as the length from the current time to failure [6]. RUL prediction results provide sufficient information support for decision-making and maintenance scheduling. The performance of RUL prediction primarily relies on the degradation modeling approaches. Generally, the RUL prediction approaches can be systematically classified into model-based methods, data-driven methods, and hybrid methods [7]. More detailed discussions about those three types of methods can be found in [8,9,10]. As one category of data-driven methods, the stochastic model-based methods have been widely used attributable to their great potential in characterizing the stochastic dynamic degradation process and providing the probability distribution of RUL [11,12]. Stochastic process models mainly include the Wiener process, the Gamma process, and the Inverse Gaussian process [13]. Among them, the Wiener process model has attached increasing interest due to its explicit statistical interpretation and illustrious mathematical properties in describing the non-monotonic degradation process [14,15,16].

In practice, the degradation process of many engineering systems occurs in a stochastic way. Thus, the RUL is a random variable, which is difficult to estimate with certainty [6]. Therefore, in the Wiener process-based RUL prediction, the commonly used approach is to estimate the probability density function (PDF) of the system RUL by modeling the sensed degradation data. Due to some unobservable internal and external factors, the degradation process of a system is often influenced by multiple sources of variability, which leads to uncertainty in RUL prediction. Typically, the degradation process is affected by three main sources of variability: temporal variability, unit-to-unit variability, and measurement variability [17]. To be specific, temporal variability depicts the inherent stochasticity of the degradation process over time, unit-to-unit variability indicates the heterogeneity of degradation trajectories among different units, and measurement variability describes the inevitable measurement error caused by noise in condition monitoring (CM) data [18]. Hence, to improve the accuracy of RUL prediction, the multi-source variability should be incorporated into the degradation modeling process simultaneously [19]. Over the past decade, various published works have described such variability in degradation modeling [20,21,22,23,24]. However, most studies only focused on the RUL estimation with one or two sources of variability, which limits their adaptivity.

Recently, for RUL prediction with three-source variability, Si et al. [19] presented a linear Wiener process-based degradation model considering the three-source variability simultaneously and derived the analytical expressions of RUL distribution. In this work, the random drift coefficient and the underlying degradation state were updated jointly via a state-space model and the Kalman filtering (KF) technique. To deal with nonlinear patterns, Zheng et al. [25] extended the work in [19] and constructed a degradation model based on the nonlinear Wiener process, integrating both nonlinearity and three-source variability into the RUL prediction. For newly developed small sample systems, to address the lack of historical data and prior information, Wang et al. [26] proposed an adaptive RUL estimation method with three-source variability based on the expectation maximization (EM) algorithm. Aiming at the unbalanced historical degradation measurements, Yu et al. [27] established a nonlinear-drift-driven prognostic model considering three-source variability and formulated the approximate analytical expressions of the RUL for both online and offline estimation scenarios. On this basis, the MLE method in conjunction with a down-sampling strategy was utilized for parameter estimation and the underflow issue averting in this study. It is noteworthy that the aforementioned linear or nonlinear Wiener process-based prognostic methods with three-source variability only focused on the single-phase degradation cases. However, in practical engineering, owing to changes in the external dynamic environment and internal degradation mechanisms, the degradation trajectories of products such as batteries [28], liquid coupling devices [29], and light emitting diodes [30] exhibit obvious two-phase characteristics with evident inflection points. Under these circumstances, the traditional single-phase model is insufficient to track the dynamics of such degradation processes. Therefore, formulating two-phase degradation models to guarantee the accuracy of RUL prediction is practical and necessary.

In recent years, researchers have reported various RUL prediction approaches considering multiple sources of variability for two-phase degradation processes. Zhang et al. [31] proposed a two-phase linear degradation model based on the Wiener process and derived the analytical forms of the RUL estimation. Chen et al. [32] extended the work in [31] and presented an extreme learning machine (ELM) algorithm to adaptively detect the random changing time of the two-phase degradation trajectory. For two-phase nonlinear degradation patterns, Lin et al. [33] formulated a nonlinear Wiener process-based degradation model and obtained an approximate analytical solution of the lifetime estimation. Hu et al. [34] constructed two RUL prediction models based on the nonlinear time-scale transformation model and the stochastic process model with purely time-dependent parameters. The commonality of the aforementioned works is that the unit-to-unit variability is considered. However, a common deficiency shared by these models is that the measurement variability is omitted. Taking into account the variability of measurement, Wang et al. [35] proposed a change-point Wiener process model with measurement errors to fit the two-phase deteriorated OLEDs and adopt the hierarchical Bayesian method to implement parameters estimation, but the number of change-points was assumed to be known a priori. Guan et al. [36] established a two-phase degradation model with measurement errors and random drift terms for RUL estimation, however, the changing time of the model was fixed, and the nonlinearity was not considered in this work. To achieve RUL prediction in the case of imperfect prior degradation information, Chai et al. [37] proposed a linear-nonlinear two-phase Wiener process-based degradation model considering measurement errors and the unknown degradation state at the change point simultaneously, which is a pioneering work. Nevertheless, the authors only described the unit-to-unit variability in parameter estimation, while ignoring the random effects of drift coefficients in the PDF expressions of RUL. In addition, the first phase of the proposed model is linear, which limits the method’s adaptability.

The above-mentioned research achieved promising results in the two-phase linear or nonlinear degradation modeling with multiple sources of variability. However, most of the existing works about two-phase patterns only considered one or two sources of variability. In contrast, the research on RUL prediction using two-phase degradation models taking into account three-source variability simultaneously is very limited. Furthermore, degrading systems exhibiting two-phase nonlinear degradation patterns are extensively encountered in practice [38,39]. Therefore, determining how to achieve two-phase nonlinear degradation modeling and RUL estimation considering three-source variability simultaneously is a compelling practical issue, which motivates our research in this paper.

To achieve this goal, several issues still need to be further investigated. First, the degradation state at the changing point of the two-phase degradation path is an unknown random variable until the changing point appears, and it is related to the changing time as well as the degradation rate of the first phase [31]. Thus, it is natural to incorporate the uncertainty of the degradation state at the changing point into RUL prediction. Currently, the state transition probability function is often applied to solve this problem in most studies [33,34]. However, these works did not consider the random degradation state at the changing point and the three-source variability simultaneously in the PDF of RUL. Second, it is noted that the changing points and the degradation rates exist difference for devices of the same batch owing to the unit-to-unit variability, and the degradation states are underlying due to the measurement noise. Therefore, determining how to realize parameter identification and real-time degradation state updating in the context of three-source variability poses an interesting challenge.

Motivated by these practical issues, the major contributions of this paper lie in the following aspects:

(1) A two-phase nonlinear Wiener process-based degradation model is formulated, where the drift coefficient and the measurement error of each phase are assumed to be random variables to describe the three-source variability.
(2) Taking into account the nonlinearity, the uncertainty of the degradation state at the changing point as well as the three-source variability simultaneously, the approximate analytical expressions of RUL estimation are derived under the concept of the first passage time (FPT).
(3) Based on the historical degradation observations of multiple units from the same batch, the offline parameter estimation is conducted by the MLE method. Subsequently, with the newly obtained degradation data of the certain operating device, the random drift coefficients and the underlying degradation states are real-time updated by combining the Bayesian rule, state-space model, and KF technique.
(4) Finally, a numerical simulation and a practical case study about the degradation data of the high-voltage pulse capacitors are implemented to verify the effectiveness and applicability of the proposed approach.

The remainder of this paper is organized as follows. In Section 2, the two-phase nonlinear Wiener process-based degradation model with three-source variability is formulated. Section 3 presents the approximate analytical solutions of the RUL estimation considering three-source variability. The model parameter estimation is conducted in Section 4. Section 5 shows the implementation details and results analysis of the experiments. The conclusions are summarized in Section 6.

## 2. Degradation Modeling Description

To describe the degradation process with two-phase nonlinearity and three-source variability, the nonlinear Wiener process is employed in our work. Inspired by the single-phase nonlinear degradation model considering three-source variability discussed in Ref. [25] and the two-phase nonlinear degradation model with unit-to-unit variability presented by Refs. [33,34], a general nonlinear Wiener process-based degradation model can be described as,

(1) $$X(t)=\left\{\begin{array}{ll} x_{0}+\lambda_{1}\int_{0}^{t}\mu_{1}(\rho ;\vartheta_{1})d\rho +\sigma_{1}B(t), & 0<t\leq \tau \\ x_{\tau }+\lambda_{2}\int_{\tau }^{t}\mu_{2}(\rho -\tau ;\vartheta_{2})d\rho +\sigma_{2}B(t-\tau ), & t>\tau \end{array}\right.$$

where X(t) denotes the actual degradation state at time t, $x_{0}$ is the initial value. For simplifying later calculation, we assume $x_{0}=0$. τ represents the changing time, thus, $x_{\tau }$ is the degradation state at the changing time. $\lambda_{1},\lambda_{2}$ and $\sigma_{1},\sigma_{2}$ represent the drift and diffusion coefficients of each phase, respectively. Meanwhile, $\mu_{1}(\rho ;\vartheta_{1})$ and $\mu_{2}(\rho -\tau ;\vartheta_{2})$ are nonlinear functions with time t and unknown parameters $\vartheta_{1},\vartheta_{2}$, which are used to describe the nonlinear characteristics of X(t). B(t) denotes the standard Brown motion. For simplicity, we assume that the abovementioned parameters are independent of phase, meanwhile, the two phases are independent of each other [33].

Owing to the dynamics of {B(t),t≥0}, the temporal variability of Equation (1) could be described. Such a modeling approach has been widely applied to characterize the stochastic degradation process of dynamic systems [27]. Further, we assume that $\lambda_{1}\sim N(\mu_{1p},\sigma_{1p}^{2})$ and $\lambda_{2}\sim N(\mu_{2p},\sigma_{2p}^{2})$ to describe the unit-to-unit variability, and they are statistically independent of {B(t),t≥0}. Moreover, $\vartheta_{1},\vartheta_{2},\sigma_{1},\sigma_{2}$ are fixed parameters used to characterize the common degradation features of all systems from the same batch. In addition, due to the influence of the dynamic environment and the non-ideal instruments, the obtained observations inevitably contain noise. Therefore, to characterize the measurement variability between observed values and the true degradation states, the degradation measurement process {Y(t),t≥0} is formulated as follows [25,37],

(2) $$Y(t)=\left\{\begin{array}{ll} X(t)+\varepsilon_{1}, & 0<t\leq \tau \\ X(t)+\varepsilon_{2}, & t>\tau \end{array}\right.$$

where $\varepsilon_{1},\varepsilon_{2}$ are the measurement errors of each phase, and assumed to be independent and identically distributed (i.i.d.) with $\varepsilon_{1}\sim N(0,\sigma_{1\varepsilon }^{2})$ and $\varepsilon_{2}\sim N(0,\sigma_{2\varepsilon }^{2})$, respectively. It is further assumed that the measurement errors are independent of X(t).

Generally, the lifetime is usually defined as the FPT when the degradation process exceeds the preset failure threshold w [40]. Based on the concept of FPT, the lifetime T of a degrading system can be defined as [19],

(3) $$T\;=\inf\left\{t:X(t)\geq w|X(0)<w\right\}$$

Then, the expression of RUL at the current time $t_{k}$ can be defined as [25]:

(4) $$L_{k}=\inf\left\{l_{k}>0:X(t_{k}+l_{k})\geq w\right\}$$

where $l_{k}$ represents the time from $t_{k}$ to the failure time, $L_{k}$ is the RUL with conditional PDF $f_{L_{k}|Y_{1:k}}(l_{k}|Y_{1:k})$, and $Y_{1:k}$ is the observations up to $t_{k}$, i.e.,$Y_{1:k}=\left\{y_{1},y_{2},\cdots ,y_{k}\right\}$.

## 3. RUL Estimation with Three-Source Variability

### 3.1. RUL Estimation with Temporal Variability and Unit-to-Unit Variability

Initially, we only consider the temporal variability in lifetime estimation. In this case, the degradation process {X(t),t≥0} described by Equation (1) could be directly observed. If the changing time and all parameters in Equation (1) are known fixed values, the PDF of the lifetime T, which only considers the temporal variability could be summarized as follows [33,34],

(1) if 0<t≤τ,

(5) $$\begin{array}{l} f_{T}(t|\lambda_{1})\\ ≅\frac{w-x_{0}-\lambda_{1}\left(\int_{0}^{t}\mu_{1}(\rho ;\vartheta_{1})d\rho -t\mu_{1}(t;\vartheta_{1})\right)}{\sqrt{2\pi \sigma_{1}^{2}t^{3}}}\exp\left[-\frac{{\left(w-x_{0}-\lambda_{1}\int_{0}^{t}\mu_{1}(\rho ;\vartheta_{1})d\rho \right)}^{2}}{2\sigma_{1}^{2}t}\right] \end{array}$$

(2) if τ<t,

(6) $$\begin{array}{l} f_{T}(t|\lambda_{2},x_{\tau })≅\frac{w-x_{\tau }-\lambda_{2}\left(\int_{\tau }^{t}\mu_{2}(\rho -\tau ;\vartheta_{2})d\rho -(t-\tau )\mu_{2}(t-\tau ;\vartheta_{2})\right)}{\sqrt{2\pi \sigma_{2}^{2}{\left(t-\tau \right)}^{3}}}\\ \times \exp\left[-\frac{{\left(w-x_{\tau }-\lambda_{2}\int_{\tau }^{t}\mu_{2}(\rho -\tau ;\vartheta_{2})d\rho \right)}^{2}}{2\sigma_{2}^{2}(t-\tau )}\right] \end{array}$$

It is noticeable that the randomness of all parameters is neglected in Equations (5) and (6). For the two-phase degradation process with a random changing time, the degradation state at the changing point is usually unknown and could be obtained through a transition probability density from $x_{0}$ to $x_{\tau }$ under an absorbable boundary w [31]. Thus, taking into account the unit-to-unit variability and the randomness of the degradation state at the changing point jointly, the PDF of the lifetime T could be obtained via the law of total probability as follows,

(7) $$f_{T}(t)=\left\{\begin{array}{ll} \int_{-\infty }^{+\infty }f_{T}(t|\lambda_{1})p(\lambda_{1})d\lambda_{1}, & 0<t\leq \tau \\ \int_{-\infty }^{w}\int_{-\infty }^{+\infty }f_{T}(t|\lambda_{2},x_{\tau })p(\lambda_{2})h_{\tau }(x_{\tau }|\lambda_{1p},\sigma_{1p})d\lambda_{2}dx_{\tau }, & t>\tau \end{array}\right.\;$$

where $h_{\tau }(x_{\tau }|\lambda_{1p},\sigma_{1p})$ is the transition probability density function considering the randomness of the first phase model, i.e., $\lambda_{1}\sim N(\lambda_{1p},\sigma_{1p}^{2})$.

Then, according to the relationship between the lifetime and RUL [19,25], the PDF of RUL that considers both temporal variability and unit-to-unit variability could be derived as follows,

**Theorem 1.**  *For the two-phase nonlinear degradation process in Equation (1) and the definition of RUL proposed in Equation (4), given the actual degradation state* $x_{k}$ *at the current time* $t_{k}$ *and* $\lambda_{1}\sim N(\lambda_{1p},\sigma_{1p}^{2}),\lambda_{2}\sim N(\lambda_{2p},\sigma_{2p}^{2})$*, the PDF of RUL with a certain changing time* τ *considering the temporal variability, unit-to-unit variability and the random degradation state at the changing point jointly can be formulated as,*

*Case 1: The current time* $t_{k}$ *is smaller than the changing time* τ *(i.e.,* $t_{k}<\tau$*)*

(8) $$f_{L}(l_{k})≅\left\{\begin{array}{ll} \begin{array}{l} \frac{1}{\sqrt{2\pi l_{k}{}^{2}\left({\left(\int_{t_{k}}^{t_{k}+l_{k}}\mu_{1}(\rho ;\vartheta_{1})d\rho \right)}^{2}\sigma_{1p}^{2}+\sigma_{1}^{2}l_{k}\right)}}\times \left[w-x_{k}-\left(\int_{t_{k}}^{t_{k}+l_{k}}\mu_{1}(\rho ;\vartheta_{1})d\rho -l_{k}\mu_{1}(t_{k}+l_{k};\vartheta_{1})\right)\right.\\ \left.\times \frac{(w-x_{k})\sigma_{1p}^{2}\int_{t_{k}}^{t_{k}+l_{k}}\mu_{1}(\rho ;\vartheta_{1})d\rho +\lambda_{1p}\sigma_{1}^{2}l_{k}}{{\left(\int_{t_{k}}^{t_{k}+l_{k}}\mu_{1}(\rho ;\vartheta_{1})d\rho \right)}^{2}\sigma_{1p}^{2}+\sigma_{1}^{2}l_{k}}\right]\times \exp\left[-\frac{{\left(w-x_{k}-\lambda_{1p}\int_{t_{k}}^{t_{k}+l_{k}}\mu_{1}(\rho ;\vartheta_{1})d\rho \right)}^{2}}{2\left({\left(\int_{t_{k}}^{t_{k}+l_{k}}\mu_{1}(\rho ;\vartheta_{1})d\rho \right)}^{2}\sigma_{1p}^{2}+\sigma_{1}^{2}l_{k}\right)}\right], \end{array} & 0<l_{k}+t_{k}\leq \tau \\ S-T, & \tau <l_{k}+t_{k} \end{array}\right.$$

*where* $S=S_{1}-S_{2}$*,* $T=T_{1}-T_{2}$*, and*

$$\begin{array}{l} S_{1}=\sqrt{\frac{r_{a2}^{2}}{2\pi {\left(t_{k}+l_{k}-\tau \right)}^{2}(\sigma_{\alpha 2}^{2}+\sigma_{\beta 2}^{2})}}\exp\left[-\frac{{\left(\lambda_{\alpha 2}-\lambda_{\beta 2}\right)}^{2}}{2(\sigma_{\alpha 2}^{2}+\sigma_{\beta 2}^{2})}\right]\\ \times \left\{\frac{\lambda_{\beta 2}\sigma_{\alpha 2}^{2}+\lambda_{\alpha 2}\sigma_{\beta 2}^{2}}{\sigma_{\alpha 2}^{2}+\sigma_{\beta 2}^{2}}\times \Phi \left(\frac{\lambda_{\beta 2}\sigma_{\alpha 2}^{2}+\lambda_{\alpha 2}\sigma_{\beta 2}^{2}}{\sqrt{\sigma_{\alpha 2}^{2}\sigma_{\beta 2}^{2}(\sigma_{\alpha 2}^{2}+\sigma_{\beta 2}^{2})}}\right)+\sqrt{\frac{\sigma_{\alpha 2}^{2}\sigma_{\beta 2}^{2}}{\sigma_{\alpha 2}^{2}+\sigma_{\beta 2}^{2}}}\times \phi \left(\frac{\lambda_{\beta 2}\sigma_{\alpha 2}^{2}+\lambda_{\alpha 2}\sigma_{\beta 2}^{2}}{\sqrt{\sigma_{\alpha 2}^{2}\sigma_{\beta 2}^{2}(\sigma_{\alpha 2}^{2}+\sigma_{\beta 2}^{2})}}\right)\right\}\\ S_{2}=\sqrt{\frac{r_{b2}^{2}}{2\pi {\left(t_{k}+l_{k}-\tau \right)}^{2}(\sigma_{\alpha 2}^{2}+\sigma_{\beta 2}^{2})}}\exp\left[-\frac{{\left(\lambda_{\alpha 2}-\lambda_{\beta 2}\right)}^{2}}{2(\sigma_{\alpha 2}^{2}+\sigma_{\beta 2}^{2})}\right]\times \left\{1-\Phi \left(-\frac{\lambda_{\beta 2}\sigma_{\alpha 2}^{2}+\lambda_{\alpha 2}\sigma_{\beta 2}^{2}}{\sqrt{\sigma_{\alpha 2}^{2}\sigma_{\beta 2}^{2}(\sigma_{\alpha 2}^{2}+\sigma_{\beta 2}^{2})}}\right)\right\}\\ T_{1}=I_{2}\times \sqrt{\frac{r_{a2}^{2}}{2\pi {\left(t_{k}+l_{k}-\tau \right)}^{2}(\sigma_{\alpha 2}^{2}+\sigma_{\beta 2}^{2})}}\exp\left[-\frac{{\left(\lambda_{\alpha 2}-\lambda_{\gamma 2}\right)}^{2}}{2(\sigma_{\alpha 2}^{2}+\sigma_{\beta 2}^{2})}\right]\\ \times \left\{\frac{\lambda_{\gamma 2}\sigma_{\alpha 2}^{2}+\lambda_{\alpha 2}\sigma_{\beta 2}^{2}}{\sigma_{\alpha 2}^{2}+\sigma_{\beta 2}^{2}}\times \Phi \left(\frac{\lambda_{\gamma 2}\sigma_{\alpha 2}^{2}+\lambda_{\alpha 2}\sigma_{\beta 2}^{2}}{\sqrt{\sigma_{\alpha 2}^{2}\sigma_{\beta 2}^{2}(\sigma_{\alpha 2}^{2}+\sigma_{\beta 2}^{2})}}\right)+\sqrt{\frac{\sigma_{\alpha 2}^{2}\sigma_{\beta 2}^{2}}{\sigma_{\alpha 2}^{2}+\sigma_{\beta 2}^{2}}}\times \phi \left(\frac{\lambda_{\gamma 2}\sigma_{\alpha 2}^{2}+\lambda_{\alpha 2}\sigma_{\beta 2}^{2}}{\sqrt{\sigma_{\alpha 2}^{2}\sigma_{\beta 2}^{2}(\sigma_{\alpha 2}^{2}+\sigma_{\beta 2}^{2})}}\right)\right\}\\ T_{2}=I_{2}\times \sqrt{\frac{r_{b2}^{2}}{2\pi {\left(t_{k}+l_{k}-\tau \right)}^{2}(\sigma_{\alpha 2}^{2}+\sigma_{\beta 2}^{2})}}\exp\left[-\frac{{\left(\lambda_{\alpha 2}-\lambda_{\gamma 2}\right)}^{2}}{2(\sigma_{\alpha 2}^{2}+\sigma_{\beta 2}^{2})}\right]\times \left\{1-\Phi \left(-\frac{\lambda_{\gamma 2}\sigma_{\alpha 2}^{2}+\lambda_{\alpha 2}\sigma_{\beta 2}^{2}}{\sqrt{\sigma_{\alpha 2}^{2}\sigma_{\beta 2}^{2}(\sigma_{\alpha 2}^{2}+\sigma_{\beta 2}^{2})}}\right)\right\}\\ \lambda_{\alpha 2}=\lambda_{2p}\int_{\tau }^{t_{k}+l_{k}}\mu_{2}(\rho -\tau ;\vartheta_{2})d\rho ,\lambda_{\beta 2}=w-x_{k}-\lambda_{1p}\int_{t_{k}}^{\tau }\mu_{1}(\rho ;\vartheta_{1})d\rho ,\\ \lambda_{\gamma 2}=-w+x_{k}-\lambda_{1p}\int_{t_{k}}^{\tau }\mu_{1}(\rho ;\vartheta_{1})d\rho -\frac{2(w-x_{k})\sigma_{1p}^{2}\int_{t_{k}}^{\tau }\mu_{1}(\rho ;\vartheta_{1})d\rho }{\sigma_{1}^{2}},\\ \sigma_{\alpha 2}^{2}=\sigma_{2p}^{2}{\left(\int_{\tau }^{t_{k}+l_{k}}\mu_{2}(\rho -\tau ;\vartheta_{2})d\rho \right)}^{2}+\sigma_{2}^{2}(t_{k}+l_{k}-\tau ),\sigma_{\beta 2}^{2}=\sigma_{1p}^{2}{\left(\int_{t_{k}}^{\tau }\mu_{1}(\rho ;\vartheta_{1})d\rho \right)}^{2}+\sigma_{1}^{2}(\tau -t_{k}),\\ r_{a2}=\frac{(t_{k}+l_{k}-\tau )\left(\sigma_{2}^{2}+\sigma_{2p}^{2}\mu_{2}(t_{k}+l_{k}-\tau ;\vartheta_{2})\int_{\tau }^{t_{k}+l_{k}}\mu_{2}(\rho -\tau ;\vartheta_{2})d\rho \right)}{\sigma_{\alpha 2}^{2}},\\ r_{b2}=\frac{(t_{k}+l_{k}-\tau )\sigma_{2}^{2}\left(\lambda_{a2}-\lambda_{2p}(t_{k}+l_{k}-\tau )\mu_{2}(t_{k}+l_{k}-\tau ;\vartheta_{2})\right)}{\sigma_{\alpha 2}^{2}},\\ I_{2}=\exp\left[\frac{2\lambda_{1p}(w-x_{k})}{\sigma_{1}^{2}}+\frac{2\left({\left(w-x_{k}\right)}^{2}\sigma_{1p}^{4}\int_{t_{k}}^{\tau }\mu_{1}(\rho ;\vartheta_{1})d\rho +{\left(w-x_{k}\right)}^{2}\sigma_{1p}^{2}\sigma_{1}^{2}\right)}{\left(\sigma_{1}^{2}+\sigma_{1p}^{2}\int_{t_{k}}^{\tau }\mu_{1}(\rho ;\vartheta_{1})d\rho \right)\sigma_{1}^{4}}\right] \end{array}$$

*Case 2: The current time* $t_{k}$ *is larger than the changing time* τ *(i.e.,* $t_{k}\geq \tau$*).*

(9) $$\begin{array}{l} f_{L}(l_{k})≅\frac{1}{\sqrt{2\pi l_{k}{}^{2}\left({\left(\int_{t_{k}}^{t_{k}+l_{k}}\mu_{2}(\rho -\tau ;\vartheta_{2})d\rho \right)}^{2}\sigma_{2p}^{2}+\sigma_{2}^{2}l_{k}\right)}}\times \left[w-x_{k}-\left(\int_{t_{k}}^{t_{k}+l_{k}}\mu_{2}(\rho -\tau ;\vartheta_{2})d\rho -l_{k}\mu_{2}(t_{k}+l_{k}-\tau ;\vartheta_{2})\right)\right.\\ \left.\times \frac{\sigma_{2p}^{2}(w-x_{k})\int_{t_{k}}^{t_{k}+l_{k}}\mu_{2}(\rho -\tau ;\vartheta_{2})d\rho +\lambda_{2p}\sigma_{2}^{2}l_{k}}{\sigma_{2p}^{2}{\left(\int_{t_{k}}^{t_{k}+l_{k}}\mu_{2}(\rho -\tau ;\vartheta_{2})d\rho \right)}^{2}+\sigma_{2}^{2}l_{k}}\right]\times \exp\left[-\frac{{\left(w-x_{k}-\lambda_{2p}\int_{t_{k}}^{t_{k}+l_{k}}\mu_{2}(\rho -\tau ;\vartheta_{2})d\rho \right)}^{2}}{2\left({\left(\int_{t_{k}}^{t_{k}+l_{k}}\mu_{2}(\rho -\tau ;\vartheta_{2})d\rho \right)}^{2}\sigma_{2p}^{2}+\sigma_{2}^{2}l_{k}\right)}\right] \end{array}$$

**Proof.**  See Appendix A. □

It is worth noting that the aforementioned results assume that the degradation state $x_{k}$ could be directly and accurately observed. However, measurement errors are inevitable in practice. Therefore, to consider the impact of measurement errors, the measurement variability should be incorporated into the RUL estimation results of Theorem 1.

### 3.2. RUL Estimation Considering Three-Source Variability

Owing to the influence of measurement errors, the true degradation state $x_{k}$ at the current time $t_{k}$ is unknown. Therefore, we assume that $x_{k}\sim N({\hat{x}}_{k|k},P_{k|k})$ and $x_{k}$ could be updated based on the degradation observations $Y_{1:k}=\left\{y_{1},y_{2},\cdots ,y_{k}\right\}$ via the updating procedure proposed in Section 4.3. Then, the PDF of RUL with three-source variability could be derived based on $x_{k}\sim N({\hat{x}}_{k|k},P_{k|k})$ and Theorem 1.

**Theorem 2.**  *For the two-phase nonlinear degradation process in Equation (1) and the definition of RUL proposed in Equation (4), given the degradation observations* $Y_{1:k}$ *up to the current time* $t_{k}$*, the PDF of RUL with a certain changing time* τ *considering the random degradation state at the changing point and the three-source variability simultaneously can be formulated as follows,*

*Case 1:* $t_{k}\leq \tau$*,*

*(1) if* $t_{k}\leq \tau$ *and* $0<l_{k}+t_{k}\leq \tau$*,*

(10) $$\begin{array}{l} f_{L_{k}|Y_{1:k}}(l_{k}|Y_{1:k})≅\frac{M_{1}-N_{1}\times \frac{\left(w-\lambda_{1p}\int_{t_{k}}^{t_{k}+l_{k}}\mu_{1}(\rho ;\vartheta_{1})d\rho \right)P_{k|k}+\upsilon_{1}(l_{k}){\hat{x}}_{k|k}}{P_{k|k}+\upsilon_{1}(l_{k})}}{\sqrt{2\pi l_{k}{}^{2}\upsilon_{1}^{2}(l_{k})\left(P_{k|k}+\upsilon_{1}(l_{k})\right)}}\\ \times \exp\left[-\frac{{\left(w-{\hat{x}}_{k|k}-\lambda_{1p}\int_{t_{k}}^{t_{k}+l_{k}}\mu_{1}(\rho ;\vartheta_{1})d\rho \right)}^{2}}{2\left(P_{k|k}+\upsilon_{1}(l_{k})\right)}\right] \end{array}$$

*where*

$$\begin{array}{l} \upsilon_{1}(l_{k})={\left(\int_{t_{k}}^{t_{k}+l_{k}}\mu_{1}(\rho ;\vartheta_{1})d\rho \right)}^{2}\sigma_{1p}^{2}+\sigma_{1}^{2}l_{k},\\ M_{1}=w\upsilon_{1}(l_{k})-\left(w\sigma_{1p}^{2}\int_{t_{k}}^{t_{k}+l_{k}}\mu_{1}(\rho ;\vartheta_{1})d\rho +\lambda_{1p}\sigma_{1}^{2}l_{k}\right)\left(\int_{t_{k}}^{t_{k}+l_{k}}\mu_{1}(\rho ;\vartheta_{1})d\rho -l_{k}\mu_{1}(t_{k}+l_{k};\vartheta_{1})\right),\\ N_{1}=\upsilon_{1}(l_{k})-\sigma_{1p}^{2}\int_{t_{k}}^{t_{k}+l_{k}}\mu_{1}(\rho ;\vartheta_{1})d\rho \left(\int_{t_{k}}^{t_{k}+l_{k}}\mu_{1}(\rho ;\vartheta_{1})d\rho -l_{k}\mu_{1}(t_{k}+l_{k};\vartheta_{1})\right) \end{array}$$

*(2) if*  $\tau <l_{k}+t_{k}$*,*

(11) $$f_{L_{k}|Y_{1:k}}(l_{k}|Y_{1:k})≅S_{new}+T_{new}$$

*where*

$$\begin{array}{l} S_{new}=\frac{1}{\sqrt{2\pi {\left(t_{k}+l_{k}-\tau \right)}^{2}(\sigma_{\alpha 2}^{2}+\sigma_{\beta 2}^{2})}}\\ \times \left\{\frac{d_{S}\exp\left[-\frac{a_{S}^{2}}{2b_{S}}\right]}{\sqrt{1+\frac{P_{k|k}}{b_{S}}}}\exp\left[\frac{2a_{S}{\hat{x}}_{k|k}+\frac{a_{S}^{2}P_{k|k}}{b_{3}}-{\hat{x}}_{k|k}^{2}}{2\left(b_{S}+P_{k|k}\right)}\right]\Phi \left(\frac{e_{S}-\sigma_{\alpha 2}^{2}{\hat{x}}_{k|k}-\frac{a_{S}\sigma_{\alpha 2}^{2}P_{k|k}}{b_{S}}+\frac{e_{S}P_{k|k}}{b_{S}}}{\sqrt{f_{S}^{2}{\left(1+\frac{P_{k|k}}{b_{S}}\right)}^{2}+\sigma_{\alpha 2}^{4}P_{k|k}\left(1+\frac{P_{k|k}}{b_{S}}\right)}}\right)\right.\\ +\frac{1}{\sqrt{2\pi P_{k|k}}}\int_{-\infty }^{+\infty }\left(\exp\left[-\frac{{\left(a_{S}-x_{k}\right)}^{2}}{2b_{S}}\right]\times \left(c_{S}x_{k}\right)\times \exp\left[-\frac{{\left(x_{k}-{\hat{x}}_{k|k}\right)}^{2}}{2P_{k|k}}\right]\times \Phi \left(\frac{e_{S}-\sigma_{\alpha 2}^{2}x_{k}}{f_{S}}\right)\right)dx_{k}\\ \left.+r_{a2}\sqrt{\frac{\sigma_{\alpha 2}^{2}\sigma_{\beta 2}^{2}}{\sigma_{\alpha 2}^{2}+\sigma_{\beta 2}^{2}}}\times \left(\frac{1}{\sqrt{2\pi }}\exp\left(-\frac{1}{2}L_{3S}\right)\times \sqrt{\frac{1}{L_{1S}^{2}P_{k|k}+1}}\exp\left[-\frac{{\left(L_{2S}-L_{1S}{\hat{x}}_{k|k}\right)}^{2}}{2\left(L_{1S}^{2}P_{k|k}+1\right)}\right]\right)\right\}\\ \sigma_{\alpha 2}^{2}=\sigma_{2p}^{2}{\left(\int_{\tau }^{t_{k}+l_{k}}\mu_{2}(\rho -\tau ;\vartheta_{2})d\rho \right)}^{2}+\sigma_{2}^{2}(t_{k}+l_{k}-\tau ),\sigma_{\beta 2}^{2}=\sigma_{1p}^{2}{\left(\int_{t_{k}}^{\tau }\mu_{1}(\rho ;\vartheta_{1})d\rho \right)}^{2}+\sigma_{1}^{2}(\tau -t_{k}),\\ r_{a2}=\frac{(t_{k}+l_{k}-\tau )\left(\sigma_{2}^{2}+\sigma_{2p}^{2}\mu_{2}(t_{k}+l_{k}-\tau ;\vartheta_{2})\int_{\tau }^{t_{k}+l_{k}}\mu_{2}(\rho -\tau ;\vartheta_{2})d\rho \right)}{\sigma_{\alpha 2}^{2}},\\ r_{b2}=\frac{(t_{k}+l_{k}-\tau )\sigma_{2}^{2}\left(\lambda_{2p}\int_{\tau }^{t_{k}+l_{k}}\mu_{2}(\rho -\tau ;\vartheta_{2})d\rho -\lambda_{2p}(t_{k}+l_{k}-\tau )\mu_{2}(t_{k}+l_{k}-\tau ;\vartheta_{2})\right)}{\sigma_{\alpha 2}^{2}},\\ a_{S}=w-\lambda_{1p}\int_{t_{k}}^{\tau }\mu_{1}(\rho ;\vartheta_{1})d\rho -\lambda_{2p}\int_{\tau }^{t_{k}+l_{k}}\mu_{2}(\rho -\tau ;\vartheta_{2})d\rho ,b_{S}=\sigma_{\alpha 2}^{2}+\sigma_{\beta 2}^{2},c_{S}=-r_{a2}\times \frac{\sigma_{\alpha 2}^{2}}{\sigma_{\alpha 2}^{2}+\sigma_{\beta 2}^{2}},\\ d_{S}=r_{a2}\times \frac{\left(w-\lambda_{1p}\int_{t_{k}}^{\tau }\mu_{1}(\rho ;\vartheta_{1})d\rho \right)\sigma_{\alpha 2}^{2}+\left(\lambda_{2p}\int_{\tau }^{t_{k}+l_{k}}\mu_{2}(\rho -\tau ;\vartheta_{2})d\rho \right)\sigma_{\beta 2}^{2}}{\sigma_{\alpha 2}^{2}+\sigma_{\beta 2}^{2}}-r_{b2},\\ e_{S}=\left(w-\lambda_{1p}\int_{t_{k}}^{\tau }\mu_{1}(\rho ;\vartheta_{1})d\rho \right)\sigma_{\alpha 2}^{2}+\left(\lambda_{2p}\int_{\tau }^{t_{k}+l_{k}}\mu_{2}(\rho -\tau ;\vartheta_{2})d\rho \right)\sigma_{\beta 2}^{2},f_{S}=\sqrt{\sigma_{\alpha 2}^{2}\sigma_{\beta 2}^{2}(\sigma_{\alpha 2}^{2}+\sigma_{\beta 2}^{2})},\\ L_{1S}=\sqrt{\frac{f_{S}^{2}+b_{S}\sigma_{\alpha 2}^{4}}{b_{S}f_{S}^{2}}},L_{2S}=\frac{a_{S}f_{S}^{2}+b_{S}e_{S}\sigma_{\alpha 2}^{4}}{\sqrt{b_{S}f_{S}^{2}\left(f_{S}^{2}+b_{S}\sigma_{\alpha 2}^{4}\right)}},L_{3S}=\frac{a_{S}^{2}f_{S}^{2}+b_{S}e_{S}^{2}}{b_{S}f_{S}^{2}}-\frac{{\left(a_{S}f_{S}^{2}+b_{S}e_{S}\sigma_{\alpha 2}^{2}\right)}^{2}}{\sqrt{b_{S}f_{S}^{2}\left(f_{S}^{2}+b_{S}\sigma_{\alpha 2}^{4}\right)}} \end{array}$$

*and*

$$\begin{array}{l} T_{new}=\frac{\exp\left[\frac{4wb_{S}\left(\lambda_{1p}\sigma_{1}^{2}+\sigma_{1p}^{2}w\right)-c_{T}^{2}\sigma_{1}^{4}}{2b_{S}\sigma_{1}^{4}}\right]}{\sqrt{2\pi {\left(t_{k}+l_{k}-\tau \right)}^{2}(\sigma_{\alpha 2}^{2}+\sigma_{\beta 2}^{2})}}\\ \times \left\{\frac{f_{T}}{\sqrt{1+\frac{D_{1T}P_{k|k}}{b_{S}}}}\exp\left[\frac{2D_{2T}{\hat{x}}_{k|k}+\frac{D_{2T}^{2}P_{k|k}}{b_{S}}-D_{1T}{\hat{x}}_{k|k}^{2}}{2\left(b_{S}+D_{1T}P_{k|k}\right)}\right]\Phi \left(\frac{g_{T}+h_{T}{\hat{x}}_{k|k}+\frac{D_{2T}h_{T}P_{k|k}}{b_{S}}+\frac{D_{1T}g_{T}P_{k|k}}{b_{S}}}{\sqrt{f_{S}^{2}{\left(1+\frac{D_{1T}P_{k|k}}{b_{S}}\right)}^{2}+h_{T}^{2}P_{k|k}\left(1+\frac{D_{1T}P_{k|k}}{b_{S}}\right)}}\right)\right.\\ +\frac{1}{\sqrt{2\pi P_{k|k}}}\int_{-\infty }^{+\infty }\left\{\exp\left[-\frac{D_{1T}}{2b_{S}}x_{k}^{2}+\frac{D_{2T}}{b_{S}}x_{k}\right]\times \left(e_{T}x_{k}\right)\times \exp\left[-\frac{{\left(x_{k}-{\hat{x}}_{k|k}\right)}^{2}}{2P_{k|k}}\right]\times \Phi \left(\frac{g_{T}+h_{T}x_{k}}{f_{S}}\right)\right\}dx_{k}\\ \left.+r_{a2}\sqrt{\frac{\sigma_{\alpha 2}^{2}\sigma_{\beta 2}^{2}}{\sigma_{\alpha 2}^{2}+\sigma_{\beta 2}^{2}}}\times \left(\frac{1}{\sqrt{2\pi }}\exp\left[-\frac{1}{2}R_{3T}\right]\times \sqrt{\frac{1}{R_{1T}^{2}P_{k|k}+1}}\exp\left[-\frac{{\left(R_{2T}-R_{1T}{\hat{x}}_{k|k}\right)}^{2}}{2\left(R_{1T}^{2}P_{k|k}+1\right)}\right]\right)\right\} \end{array}$$

$$\begin{array}{l} a_{T}=\frac{2\sigma_{1p}^{2}}{\sigma_{1}^{4}},b_{T}=\frac{2\lambda_{1p}\sigma_{1}^{2}+4w\sigma_{1p}^{2}}{\sigma_{1}^{4}},c_{T}=w+\lambda_{1p}\int_{t_{k}}^{\tau }\mu_{1}(\rho ;\vartheta_{1})d\rho +\lambda_{2p}\int_{\tau }^{t_{k}+l_{k}}\mu_{2}(\rho -\tau ;\vartheta_{2})d\rho +\frac{2w\sigma_{1p}^{2}\int_{t_{k}}^{\tau }\mu_{1}(\rho ;\vartheta_{1})d\rho }{\sigma_{1}^{2}}\\ d_{T}=\frac{\sigma_{1}^{2}+2\sigma_{1p}^{2}\int_{t_{k}}^{\tau }\mu_{1}(\rho ;\vartheta_{1})d\rho }{\sigma_{1}^{2}},D_{1T}=d_{T}^{2}-2a_{T}b_{S},D_{2T}=c_{T}d_{T}-b_{S}b_{T},e_{T}=\frac{r_{a2}\sigma_{\alpha 2}^{2}\left(\sigma_{1}^{2}+2\sigma_{1p}^{2}\int_{t_{k}}^{\tau }\mu_{1}(\rho ;\vartheta_{1})d\rho \right)}{\sigma_{1}^{2}\left(\sigma_{\alpha 2}^{2}+\sigma_{\beta 2}^{2}\right)},\\ f_{T}=r_{a2}\times \frac{\left(-w-\lambda_{1p}\int_{t_{k}}^{\tau }\mu_{1}(\rho ;\vartheta_{1})d\rho -\frac{2w\sigma_{1p}^{2}\int_{t_{k}}^{\tau }\mu_{1}(\rho ;\vartheta_{1})d\rho }{\sigma_{1}^{2}}\right)\sigma_{\alpha 2}^{2}+\lambda_{2p}\sigma_{\beta 2}^{2}\int_{\tau }^{t_{k}+l_{k}}\mu_{2}(\rho -\tau ;\vartheta_{2})d\rho }{\sigma_{\alpha 2}^{2}+\sigma_{\beta 2}^{2}}-r_{b2},\\ g_{T}=\left(-w-\lambda_{1p}\int_{t_{k}}^{\tau }\mu_{1}(\rho ;\vartheta_{1})d\rho -\frac{2w\sigma_{1p}^{2}\int_{t_{k}}^{\tau }\mu_{1}(\rho ;\vartheta_{1})d\rho }{\sigma_{1}^{2}}\right)\sigma_{\alpha 2}^{2}+\lambda_{2p}\sigma_{\beta 2}^{2}\int_{\tau }^{t_{k}+l_{k}}\mu_{2}(\rho -\tau ;\vartheta_{2})d\rho ,\\ h_{T}=\frac{\sigma_{\alpha 2}^{2}\left(\sigma_{1}^{2}+2\sigma_{1p}^{2}\int_{t_{k}}^{\tau }\mu_{1}(\rho ;\vartheta_{1})d\rho \right)}{\sigma_{1}^{2}},R_{1T}=\sqrt{\frac{D_{1T}f_{S}^{2}+b_{S}h_{T}^{2}}{b_{S}f_{S}^{2}}},R_{2T}=\frac{D_{2T}f_{S}^{2}-b_{S}g_{T}h_{T}}{\sqrt{b_{S}f_{S}^{2}\left(D_{1T}f_{S}^{2}+b_{S}h_{T}^{2}\right)}},R_{3T}=\frac{g_{T}^{2}}{f_{S}^{2}}-\frac{{\left(D_{2T}f_{S}^{2}-b_{S}g_{T}h_{T}\right)}^{2}}{b_{S}f_{S}^{2}\left(D_{1T}f_{S}^{2}+b_{S}h_{T}^{2}\right)} \end{array}$$

*Case 2: if* $\tau <t_{k}$*,*

(12) $$\begin{array}{l} f_{L_{k}|Y_{\tilde{\tau }:k}}(l_{k}|Y_{\tilde{\tau }:k})\\ =\frac{M_{2}-N_{2}\times \frac{\left(w-\lambda_{2p}\int_{t_{k}}^{t_{k}+l_{k}}\mu_{2}(\rho -\tau ;\vartheta_{2})d\rho \right)P_{k|k}+\upsilon_{2}(l_{k}){\hat{x}}_{k|k}}{P_{k|k}+\upsilon_{2}(l_{k})}}{\sqrt{2\pi l_{k}{}^{2}\upsilon_{2}^{2}(l_{k})\left(P_{k|k}+\upsilon_{2}(l_{k})\right)}}\times \exp\left[-\frac{{\left(w-{\hat{x}}_{k|k}-\lambda_{2p}\int_{t_{k}}^{t_{k}+l_{k}}\mu_{2}(\rho -\tau ;\vartheta_{2})d\rho \right)}^{2}}{2\left(P_{k|k}+\upsilon_{2}(l_{k})\right)}\right] \end{array}$$

*where*

$$\begin{array}{l} \upsilon_{2}(l_{k})={\left(\int_{t_{k}}^{t_{k}+l_{k}}\mu_{2}(\rho -\tau ;\vartheta_{2})d\rho \right)}^{2}\sigma_{2p}^{2}+\sigma_{2}^{2}l_{k},\\ M_{2}=w\upsilon_{2}(l_{k})-\left(w\sigma_{2p}^{2}\int_{t_{k}}^{t_{k}+l_{k}}\mu_{2}(\rho -\tau ;\vartheta_{2})d\rho +\lambda_{2p}\sigma_{2}^{2}l_{k}\right)\left(\int_{t_{k}}^{t_{k}+l_{k}}\mu_{2}(\rho -\tau ;\vartheta_{2})d\rho -l_{k}\mu_{2}(t_{k}+l_{k}-\tau ;\vartheta_{2})\right)\\ N_{2}=\upsilon_{2}(l_{k})-\sigma_{2p}^{2}\int_{t_{k}}^{t_{k}+l_{k}}\mu_{2}(\rho -\tau ;\vartheta_{2})d\rho \left(\int_{t_{k}}^{t_{k}+l_{k}}\mu_{2}(\rho -\tau ;\vartheta_{2})d\rho -l_{k}\mu_{2}(t_{k}+l_{k}-\tau ;\vartheta_{2})\right) \end{array}.$$

**Proof.**  See Appendix B. □

In addition, it is worth mentioning that the changing time of the above results is a fixed value. In the case of three-source variability, considering the randomness of the changing point τ, the PDF of RUL could be derived as follows [31],

(13) $$\;f_{L}(l_{k})=\int_{t_{k}}^{+\infty }f_{L}(l_{k}|\tau )p(\tau )d\tau$$

where p(τ) represents the PDF of the changing time τ. Equation (13) could be solved by some numerical methods.

## 4. Model Parameter and Degradation State Estimation

Before conducting RUL prediction, the unknown model parameters should be identified first. Considering the common characteristics of devices from the same batch and the individual features of the specific operating device, the offline and online methods are adopted jointly to identify the model parameters. Firstly, based on the historical observations of devices within the same batch, the MLE method is used for offline parameter estimation. Then, the changing point detection method is constructed on this basis. Finally, according to the real-time monitoring data of one specific operating device, the online updating procedures of the model parameters are realized via the Bayesian rule and KF algorithm.

### 4.1. Offline Parameter Estimation

In this subsection, we mainly focus on the estimation of the unknown model parameters, which are denoted as $\Theta =\left\{\lambda_{1p},\sigma_{1p},\vartheta_{1},\sigma_{1},\sigma_{1\varepsilon },\lambda_{2p},\sigma_{2p},\vartheta_{2},\sigma_{2},\sigma_{2\varepsilon }\right\}$. Among them, $\lambda_{1}\sim N(\lambda_{1p},\sigma_{1p}^{2}),\lambda_{2}\sim N(\lambda_{2p},\sigma_{2p}^{2})$ are random variables that describe the unit-to-unit variability, and $\vartheta_{1},\sigma_{1},\sigma_{1\varepsilon },\vartheta_{2},\sigma_{2},\sigma_{2\varepsilon }$ are deterministic parameters that depict the common degradation features of devices from the same batch. It is noted that τ will be estimated via the changing point detection method in Section 4.2.

It is assumed that the historical observations of N devices within the same batch are known, i.e., $Y_{1:N}=\{Y_{1},Y_{2},\cdots ,Y_{N}\}$, and the corresponding actual degradation state data is denoted as $X_{1:N}=\{X_{1},X_{2},\cdots ,X_{N}\}$. The observations $Y_{n}=\{y_{n,0},y_{n,1},\cdots ,y_{n,m_{n}}\}$ of the n-th device are measured at time $\left\{t_{n,0},t_{n,1},\cdots ,t_{n,m_{n}}\right\}$ and $m_{n}$ denotes the available number of the observations. We define that the measured increment of the *n*-*th* device is $\Delta y_{n,j}=y_{n,j}-y_{n,j-1}$, where $j=1,2,\cdots ,m_{n}$. Thus, $\Delta Y_{n}=\{\Delta y_{n,1},\Delta y_{n,2},\cdots ,\Delta y_{n,m_{n}}\}$. For simplicity, the time interval is assumed to be a constant, i.e., $\Delta t=t_{n,j}-t_{n,j-1}$. Suppose $\tau_{n}$ denotes the changing time of the *n*-*th* device, and it is assumed that ${\tilde{\tau }}_{n}=\tau_{n}/\Delta t\in \{0,1,\ldots ,m_{n}\}$ denotes the changing point location for simplifying the later calculation. Scilicet, the changing time $\tau_{n}$ only appears at the measurement time $\left\{t_{n,0},t_{n,1},\cdots ,t_{n,m_{n}}\right\}$. As a result, $\Delta Y_{1n}=\{\Delta y_{n,1},\Delta y_{n,2},\cdots ,\Delta y_{n,{\tilde{\tau }}_{n}}\}$ represents the measured increments in the first phase, and $\Delta Y_{2n}=\{\Delta y_{n,{\tilde{\tau }}_{n}+1},\Delta y_{n,{\tilde{\tau }}_{n}+2},\cdots ,\Delta y_{n,m_{n}}\}$ represents the measured increments in the second phase.

According to the definition in Equation (1) and the properties of the Wiener process, the measured increments $\Delta Y_{n}=\{\Delta y_{n,1},\Delta y_{n,2},\cdots ,\Delta y_{n,m_{n}}\}$ follow the multivariate normal distribution with expectation and covariance matrix as follows,

if $0<t_{n,j}\leq \tau_{n}$,

(14) $$E\left[\Delta Y_{1n}\right]=\lambda_{1p}\Delta T_{1n},\Sigma_{1n}=\sigma_{1p}^{2}\Delta T_{1n}\Delta {T^{\prime }}_{1n}+\Omega_{1n}$$

if $t_{n,j}>\tau_{n}$,

(15) $$E\left[\Delta Y_{2n}\right]=\lambda_{2p}\Delta T_{2n},\Sigma_{2n}=\sigma_{2p}^{2}\Delta T_{2n}\Delta {T^{\prime }}_{2n}+\Omega_{2n}$$

where $\Omega_{1n}=\sigma_{1}^{2}D_{1n}+\sigma_{1\varepsilon }^{2}P_{1n},\Omega_{2n}=\sigma_{2}^{2}D_{2n}+\sigma_{2\varepsilon }^{2}P_{2n}$,

$$\begin{array}{l} P_{1n}={\left[\begin{array}{ccccc} 1 & -1 & 0 & \cdots & 0\\ -1 & 2 & -1 & \cdots & \vdots \\ 0 & -1 & 2 & \ddots & 0\\ \vdots & \vdots & \ddots & \ddots & -1\\ 0 & 0 & \cdots & -1 & 2 \end{array}\right]}_{{\tilde{\tau }}_{n}\times {\tilde{\tau }}_{n}},P_{2n}={\left[\begin{array}{ccccc} 1 & -1 & 0 & \cdots & 0\\ -1 & 2 & -1 & \cdots & \vdots \\ 0 & -1 & 2 & \ddots & 0\\ \vdots & \vdots & \ddots & \ddots & -1\\ 0 & 0 & \cdots & -1 & 2 \end{array}\right]}_{\left(m_{n}-{\tilde{\tau }}_{n})\times (m_{n}-{\tilde{\tau }}_{n}\right)}\\ \Delta T_{1n}={\left[\int_{0}^{t_{n,1}}\mu_{1}(\rho ;\vartheta_{1})d\rho ,\int_{t_{n,1}}^{t_{n,2}}\mu_{1}(\rho ;\vartheta_{1})d\rho ,\ldots ,\int_{t_{n,{\tilde{\tau }}_{n}-1}}^{t_{n,{\tilde{\tau }}_{n}}}\mu_{1}(\rho ;\vartheta_{1})d\rho \right]}^{\prime }\\ \Delta T_{2n}={\left[\int_{t_{n,{\tilde{\tau }}_{n}}}^{t_{n,{\tilde{\tau }}_{n}+1}}\mu_{2}(\rho -\tau ;\vartheta_{2})d\rho ,\int_{t_{n,{\tilde{\tau }}_{n}+1}}^{t_{n,{\tilde{\tau }}_{n}+2}}\mu_{2}(\rho -\tau ;\vartheta_{2})d\rho ,\ldots ,\int_{t_{n,m_{n-1}}}^{t_{n,m_{n}}}\mu_{2}(\rho -\tau ;\vartheta_{2})d\rho \right]}^{\prime }\\ D_{1n}=diag(\Delta t_{n,1},\Delta t_{n,2},\ldots ,\Delta t_{n,\tau_{n}}),D_{2n}=diag(\Delta t_{n,{\tilde{\tau }}_{n+1}},\Delta t_{n,{\tilde{\tau }}_{n+2}},\ldots ,\Delta t_{n,m_{n}}) \end{array}$$

Thus, the log-likelihood function of Θ could be expressed as follows,

(16) $$\begin{array}{l} \ln(\Theta |Y_{1:N})\\ =-\frac{\ln(2\pi )}{2}\sum_{n=1}^{N}{\tilde{\tau }}_{n}-\frac{1}{2}\sum_{n=1}^{N}\ln|\Sigma_{1n}|-\frac{1}{2}\sum_{n=1}^{N}(\Delta Y_{1n}-\lambda_{1p}\Delta T_{1n}{)}^{\prime }\Sigma_{1n}^{-1}(\Delta Y_{1n}-\lambda_{1p}\Delta T_{1n})\\ -\frac{\ln(2\pi )}{2}\sum_{n=1}^{N}(m_{n}-{\tilde{\tau }}_{n})-\frac{1}{2}\sum_{n=1}^{N}\ln|\Sigma_{2n}|-\frac{1}{2}\sum_{n=1}^{N}(\Delta Y_{2n}-\lambda_{2p}\Delta T_{2n}{)}^{\prime }\Sigma_{2n}^{-1}(\Delta Y_{2n}-\lambda_{2p}\Delta T_{2n}) \end{array}$$

To facilitate calculation, let ${\tilde{\sigma }}_{1}^{2}=\sigma_{1}^{2}/\sigma_{1p}^{2},{\tilde{\sigma }}_{1\varepsilon }^{2}=\sigma_{1\varepsilon }^{2}/\sigma_{1p}^{2},{\tilde{\Sigma }}_{1n}=\Sigma_{1n}/\sigma_{1p}^{2}$, and ${\tilde{\sigma }}_{2}^{2}=\sigma_{2}^{2}/\sigma_{2p}^{2},{\tilde{\sigma }}_{2\varepsilon }^{2}=\sigma_{2\varepsilon }^{2}/\sigma_{2p}^{2},{\tilde{\Sigma }}_{2n}=\Sigma_{2n}/\sigma_{2p}^{2}$. Then, the log-likelihood function in Equation (16) could be written as,

(17) $$\begin{array}{l} \ln(\Theta |Y_{1:N})=-\frac{\ln(2\pi )}{2}\sum_{n=1}^{N}{\tilde{\tau }}_{n}-\frac{\ln\sigma_{1p}^{2}}{2}\sum_{n=1}^{N}{\tilde{\tau }}_{n}-\frac{1}{2}\sum_{n=1}^{N}\ln|{\tilde{\Sigma }}_{1n}|-\frac{1}{2\sigma_{1p}^{2}}\sum_{n=1}^{N}(\Delta Y_{1n}-\lambda_{1p}\Delta T_{1n}{)}^{\prime }{\tilde{\Sigma }}_{1n}^{-1}(\Delta Y_{1n}-\lambda_{1p}\Delta T_{1n})\\ -\frac{\ln(2\pi )}{2}\sum_{n=1}^{N}\left(m_{n}-{\tilde{\tau }}_{n}\right)-\frac{\ln\sigma_{2p}^{2}}{2}\sum_{n=1}^{N}(m_{n}-{\tilde{\tau }}_{n})-\frac{1}{2}\sum_{n=1}^{N}\ln|{\tilde{\Sigma }}_{2n}|-\frac{1}{2\sigma_{2p}^{2}}\sum_{n=1}^{N}(\Delta Y_{2n}-\lambda_{2p}\Delta T_{2n}{)}^{\prime }{\tilde{\Sigma }}_{2n}^{-1}(\Delta Y_{2n}-\lambda_{2p}\Delta T_{2n}) \end{array}$$

Under the framework of the MLE algorithm, taking the first partial derivatives of $\ln(\Theta |Y_{1:N})$ with respect to $\lambda_{1p}$ and $\sigma_{1p}^{2}$, respectively. Then, the MLE of $\lambda_{1p},\sigma_{1p}^{2}$ could be obtained by setting these two derivatives to zero.

(18) $${\hat{\lambda }}_{1p}=\frac{\sum_{n=1}^{N}\Delta {T^{\prime }}_{1n}{\tilde{\Sigma }}_{1n}^{-1}\Delta Y_{1n}}{\sum_{n=1}^{N}\Delta {T^{\prime }}_{1n}{\tilde{\Sigma }}_{1n}^{-1}\Delta T_{1n}},{\hat{\sigma }}_{1p}^{2}=\frac{\sum_{n=1}^{N}(\Delta Y_{1n}-{\hat{\lambda }}_{1p}\Delta T_{1n}{)}^{\prime }{\tilde{\Sigma }}_{1n}^{-1}(\Delta Y_{1n}-{\hat{\lambda }}_{1p}\Delta T_{1n})}{\sum_{n=1}^{N}{\tilde{\tau }}_{n}}$$

Similarly, the MLE of $\lambda_{2p},\sigma_{2p}^{2}$ could be obtained as,

(19) $${\hat{\lambda }}_{2p}=\frac{\sum_{n=1}^{N}\Delta {T^{\prime }}_{2n}{\tilde{\Sigma }}_{2n}^{-1}\Delta Y_{2n}}{\sum_{n=1}^{N}\Delta {T^{\prime }}_{2n}{\tilde{\Sigma }}_{2n}^{-1}\Delta T_{2n}},{\hat{\sigma }}_{2p}^{2}=\frac{\sum_{n=1}^{N}(\Delta Y_{2n}-{\hat{\lambda }}_{2p}\Delta T_{2n}{)}^{\prime }{\tilde{\Sigma }}_{2n}^{-1}(\Delta Y_{2n}-{\hat{\lambda }}_{2p}\Delta T_{2n})}{\sum_{n=1}^{N}\left(m_{n}-{\tilde{\tau }}_{n}\right)}$$

However, the results of ${\hat{\lambda }}_{1p},{\hat{\sigma }}_{1p}^{2}$ and ${\hat{\lambda }}_{2p},{\hat{\sigma }}_{2p}^{2}$ still contain the unknown parameters, i.e., $\vartheta_{1},{\tilde{\sigma }}_{1}^{2},{\tilde{\sigma }}_{1\varepsilon }^{2}$ and $\vartheta_{2},{\tilde{\sigma }}_{2}^{2},{\tilde{\sigma }}_{2\varepsilon }^{2}$. Fortunately, the MLE of these parameters could be calculated based on ${\hat{\lambda }}_{1p},{\hat{\sigma }}_{1p}^{2}$ and ${\hat{\lambda }}_{2p},{\hat{\sigma }}_{2p}^{2}$.

Since the two phases are independent of each other, thus, substituting Equation (18) into Equation (17), the profile likelihood function of $\vartheta_{1},{\tilde{\sigma }}_{1}^{2},{\tilde{\sigma }}_{1\varepsilon }^{2}$ according to ${\hat{\lambda }}_{1p},{\hat{\sigma }}_{1p}^{2}$ could be expressed as follows,

(20) $$\ln L(\vartheta_{1},{\tilde{\sigma }}_{1}^{2},{\tilde{\sigma }}_{1\varepsilon }^{2}|\Delta Y_{1n})=-\frac{\ln(2\pi )}{2}\sum_{n=1}^{N}{\tilde{\tau }}_{n}-\frac{\ln{\hat{\sigma }}_{1p}^{2}}{2}\sum_{n=1}^{N}{\tilde{\tau }}_{n}-\frac{1}{2}\sum_{n=1}^{N}{\tilde{\tau }}_{n}-\frac{1}{2}\sum_{n=1}^{N}\ln|{\tilde{\Sigma }}_{1n}|$$

Similarly, by substituting Equation (19) into Equation (17), the profile likelihood function of $\vartheta_{2},{\tilde{\sigma }}_{2}^{2},{\tilde{\sigma }}_{2\varepsilon }^{2}$ according to ${\hat{\lambda }}_{2p},{\hat{\sigma }}_{2p}^{2}$ could be e formulated as follows.

(21) $$\begin{array}{l} \ln L(\vartheta_{2},{\tilde{\sigma }}_{2}^{2},{\tilde{\sigma }}_{2\varepsilon }^{2}|\Delta Y_{2n})\\ =-\frac{\ln(2\pi )}{2}\sum_{n=1}^{N}\left(m_{n}-{\tilde{\tau }}_{n}\right)-\frac{\ln{\hat{\sigma }}_{2p}^{2}}{2}\sum_{n=1}^{N}\left(m_{n}-{\tilde{\tau }}_{n}\right)-\frac{1}{2}\sum_{n=1}^{N}\left(m_{n}-{\tilde{\tau }}_{n}\right)-\frac{1}{2}\sum_{n=1}^{N}\ln|{\tilde{\Sigma }}_{2n}| \end{array}$$

The MLE of $\vartheta_{1},{\tilde{\sigma }}_{1}^{2},{\tilde{\sigma }}_{1\varepsilon }^{2}$ and $\vartheta_{2},{\tilde{\sigma }}_{2}^{2},{\tilde{\sigma }}_{2\varepsilon }^{2}$ could be obtained through a multi-dimensional search, which is implemented by the “fminsearch” function in MATLAB.

Then, substituting the MLE of $\vartheta_{1},{\tilde{\sigma }}_{1}^{2},{\tilde{\sigma }}_{1\varepsilon }^{2}$ and $\vartheta_{2},{\tilde{\sigma }}_{2}^{2},{\tilde{\sigma }}_{2\varepsilon }^{2}$ into Equations (18) and (19), respectively, the final estimates of $\lambda_{1p},\sigma_{1p}^{2}$ and $\lambda_{2p},\sigma_{2p}^{2}$ could be obtained. Then, the MLE of $\vartheta_{1},\sigma_{1},\sigma_{1\varepsilon }$ and $\vartheta_{2},\sigma_{2},\sigma_{2\varepsilon }$ could be calculated via inverting the relations ${\tilde{\sigma }}_{1}^{2}=\sigma_{1}^{2}/\sigma_{1p}^{2},{\tilde{\sigma }}_{1\varepsilon }^{2}=\sigma_{1\varepsilon }^{2}/\sigma_{1p}^{2},{\tilde{\Sigma }}_{1n}=\Sigma_{1n}/\sigma_{1p}^{2}$ and ${\tilde{\sigma }}_{2}^{2}=\sigma_{2}^{2}/\sigma_{2p}^{2},{\tilde{\sigma }}_{2\varepsilon }^{2}=\sigma_{2\varepsilon }^{2}/\sigma_{2p}^{2},{\tilde{\Sigma }}_{2n}=\Sigma_{2n}/\sigma_{2p}^{2}$, accordingly.

### 4.2. Changing Point Detection

For the two-phase nonlinear degradation process, changing point detection is crucial for parameter estimation and RUL prediction. It is assumed that the changing time is a random variable and follows the normal distribution, i.e., $\tau ∼N(\mu_{\tau },\sigma_{\tau }^{2})$. Then, for a specific operating device, the log-likelihood function of τ can be formulated as follows,

(22) $$\begin{array}{l} \ln L(\Theta |\Delta Y_{n})=-\frac{{\tilde{\tau }}_{n}}{2}\ln(2\pi )-\frac{1}{2}\ln|\Sigma_{1n}|-\frac{1}{2}(\Delta Y_{1n}-\lambda_{1p}\Delta T_{1n}{)}^{\prime }\Sigma_{1n}^{-1}(\Delta Y_{1n}-\lambda_{1p}\Delta T_{1n})\\ -\frac{m_{n}-{\tilde{\tau }}_{n}}{2}\ln(2\pi )-\frac{1}{2}\ln|\Sigma_{2n}|-\frac{1}{2}(\Delta Y_{2n}-\lambda_{2p}\Delta T_{2n}{)}^{\prime }\Sigma_{2n}^{-1}(\Delta Y_{2n}-\lambda_{2p}\Delta T_{2n}) \end{array}$$

where $\Delta Y_{n}=(\Delta y_{n,1},\Delta y_{n,2},\cdots ,\Delta y_{n,k}{)}^{\prime }$.

Similar to the offline parameter estimation process, the MLE of the deterministic parameters and the expressions of ${\hat{\lambda }}_{1p},{\hat{\sigma }}_{1p}^{2},{\hat{\lambda }}_{2p},{\hat{\sigma }}_{2p}^{2}$ could be derived. Then, by substituting these MLE values and expressions into Equation (22), a log-likelihood function that is only related to the changing point location ${\tilde{\tau }}_{n}$ can be formulated. Thus, the optimal changing time ${\hat{\tau }}_{n}$ of the *n*-*th* device could be expressed as follows,

(23) $${\hat{\tau }}_{n}=\Delta t\times \underset{{\tilde{\tau }}_{n}}{\arg\max}\ln L({\tilde{\tau }}_{n}|\Delta Y_{n})$$

On this basis, maximizing $\ln L({\tilde{\tau }}_{n}|X_{n})$ by enumerating all possible values of ${\tilde{\tau }}_{n}$, the optimal changing time ${\hat{\tau }}_{n}$ could be obtained.

In addition, for a specific operating device, if $t_{k}>{\hat{\tau }}_{n}$ at the current time $t_{k}$, the changing time has appeared and is a certain value. However, if $t_{k}<{\hat{\tau }}_{n}$, which means that the changing point has not appeared and is an unknown random variable. Consequently, we need to update its distribution. In this case, the above-estimated value ${\hat{\tau }}_{n}$ can be treated as the observations of the changing time. Then, the mean and variance of τ could be formulated as [33],

(24) $$\mu_{\tau }=\frac{1}{N}\sum_{n=1}^{N}{\hat{\tau }}_{n},\sigma_{\tau }=\sqrt{\frac{1}{N}\sum_{n=1}^{N}{\left({\hat{\tau }}_{n}-\mu_{\tau }\right)}^{2}}$$

### 4.3. Online Implicit State Updating

For the RUL estimation with three-source variability, the random drift coefficients $\lambda_{1},\lambda_{2}$ and the underlying degradation state $x_{k}$ need to be updated jointly based on the observations. In practice, the posterior estimates of the drift coefficients have little effect with $x_{k}$ [24]. Without loss of generality, it is assumed that they are independent of each other. On this basis, the update mechanism of implicit states could be conducted through two steps: firstly, the random drift coefficients are updated by the Bayesian method, and then the KF algorithm is adopted to update $x_{k}$.

As to a specific operating device, if the current time is $t_{k}$, the degradation observation is denoted as $Y_{1:k}=\left\{y_{1},y_{2},\cdots ,y_{k}\right\}$, and the corresponding actual degradation state is denoted as $X_{1:k}=\left\{x_{1},x_{2},\cdots ,x_{k}\right\}$. It is defined that the observation increment is $\Delta y_{j}=y_{j}-y_{j-1}$, and the time interval is $\Delta t_{j}=t_{j}-t_{j-1},j=1,\cdots ,k$. If $t_{k}\leq \tau$, all the observations $Y_{1:k}=\left\{y_{1},y_{2},\cdots ,y_{k}\right\}$ can be used for model updating, thus, $\Delta Y_{k}=\left\{\Delta y_{1},\Delta y_{2},\cdots ,\Delta y_{k}\right\}$. Meanwhile, let $\Delta T_{k}=\int_{t_{k-1}}^{t_{k}}\mu_{1}(\rho ;\vartheta_{1})d\rho$, and we have $\Delta T_{k}=\left\{\Delta T_{1},\Delta T_{2},\cdots ,\Delta T_{k}\right\}$. Whereas if $t_{k}>\tau$, only the observations in the second phase could be applied, i.e., $Y_{\tilde{\tau }:k}=\left\{y_{\tilde{\tau }},y_{\tilde{\tau }+1},\cdots ,y_{k}\right\}$, thus, $\Delta Y_{k}=\left\{\Delta y_{\tilde{\tau }+1},\Delta y_{\tilde{\tau }+2},\cdots ,\Delta y_{k}\right\}$. Let $\Delta T_{k}=\int_{t_{k-1}}^{t_{k}}\mu_{2}(\rho -\tau ;\vartheta_{2})d\rho$, and consequently, $\Delta T_{k}=\left\{\Delta T_{\tilde{\tau }+1},\Delta T_{\tilde{\tau }+2},\cdots ,\Delta T_{k}\right\}$.

To update the drift coefficients, let $\lambda_{1p,0},\sigma_{1p,0}$ and $\lambda_{2p,0},\sigma_{2p,0}$ attained in Section 4.1 represent the prior values of $\lambda_{1}$ and $\lambda_{2}$, respectively. Then, according to the Bayesian rule [24], the following results could be obtained,

if $t_{k}\leq \tau$,

(25) $$\lambda_{1}|Y_{1:k}∼N(\lambda_{1p,k},\sigma_{1p,k}^{2})$$

where

$$\lambda_{1p,k}=\frac{\Delta {T^{\prime }}_{k}\Omega_{k}^{-1}\Delta Y_{k}\sigma_{1p,0}^{2}+\lambda_{1p,0}}{\Delta {T^{\prime }}_{k}\Omega_{k}^{-1}\Delta T_{k}\cdot \sigma_{1p,0}^{2}+1},\sigma_{1p,k}^{2}=\frac{\sigma_{1p,0}^{2}}{\Delta {T^{\prime }}_{k}\Omega_{k}^{-1}\Delta T_{k}\cdot \sigma_{1p,0}^{2}+1}$$

if $t_{k}>\tau$,

(26) $$\lambda_{2}|Y_{\tilde{\tau }:k}∼N(\lambda_{2p,k},\sigma_{2p,k}^{2})$$

where

$$\lambda_{2p,k}=\frac{\Delta {T^{\prime }}_{k}\Omega_{k}^{-1}\Delta Y_{k}\cdot \sigma_{2p,0}^{2}+\lambda_{2p,0}}{\Delta {T^{\prime }}_{k}\Omega_{k}^{-1}\Delta T_{k}\cdot \sigma_{2p,0}^{2}+1},\sigma_{2p,k}^{2}=\frac{\sigma_{2p,0}^{2}}{\Delta {T^{\prime }}_{k}\Omega_{k}^{-1}\Delta T_{k}\cdot \sigma_{2p,0}^{2}+1}$$

To update the underlying degradation state, i.e., $x_{k}\sim N({\hat{x}}_{k|k},P_{k|k})$, the state and measurement formulas in Equations (1) and (2) should be converted into discrete time equations. In this case, a general state space model is constructed as follows,

(27) $$\left\{\begin{array}{l} x_{k}=x_{k-1}+\lambda_{k-1}\Delta T_{k}+v_{k}\\ y_{k}=x_{k}+\varepsilon_{sk} \end{array}\right.$$

where s=1,2 denotes the phase. If $t_{k}\leq \tau$, thus, s=1, $v_{k}=\sigma_{1}\left(B(t_{k})-B(t_{k-1})\right)$, $v_{k}\sim N(0,\sigma_{1}^{2}\Delta t_{k})$, and $\varepsilon_{k}\sim N(0,\sigma_{1\varepsilon }^{2})$. Whereas if $t_{k}>\tau$, we have s=2, $v_{k}=\sigma_{2}\left(B(t_{k}-\tau )-B(t_{k-1}-\tau )\right)$, $v_{k}\sim N(0,\sigma_{2}^{2}\Delta t_{k})$, and $\varepsilon_{k}\sim N(0,\sigma_{2\varepsilon }^{2})$. In addition, it is assumed that ${\left\{v_{k}\right\}}_{k\geq 1}$ and ${\left\{\varepsilon_{sk}\right\}}_{k\geq 1}$ are independent of each other.

The KF algorithm is adopted to solve Equation (27). For $x_{k}\sim N({\hat{x}}_{k|k},P_{k|k})$, if $t_{k}\leq \tau$, we define ${\hat{x}}_{k|k}=E\left[x_{k}|Y_{1:k},\Theta \right]$ and $P_{k|k}=\mathrm{var}\left[x_{k}|Y_{1:k},\Theta \right]$, if $t_{k}>\tau$, we define ${\hat{x}}_{k|k}=E\left[x_{k}|Y_{\tilde{\tau }+1:k},\Theta \right]$, $P_{k|k}=\mathrm{var}\left[x_{k}|Y_{\tilde{\tau }+1:k},\Theta \right]$. Then, the KF process could be summarized as follows,

State estimation:

$$\begin{array}{l} {\hat{x}}_{k|k-1}={\hat{x}}_{k-1|k-1}+\lambda_{sp,k-1}\Delta T_{k}\\ {\hat{x}}_{k|k}={\hat{x}}_{k|k-1}+K(k)(y_{k}-{\hat{x}}_{k|k-1})\\ K(k)=P_{k|k-1}{\left(P_{k|k-1}+\sigma_{s\varepsilon }^{2}\right)}^{-1}\\ P_{k|k-1}=P_{k-1|k-1}+\sigma_{s}^{2}(t_{k}-t_{k-1}) \end{array}$$

Variance update:

(28) $$P_{k|k}=\left(1-K(k)\right)P_{k|k-1}$$

where s=1,2 denotes the phase, when s=1, $\lambda_{1p,k-1}$ could be obtained based on Equation (25), when s=2, $\lambda_{2p,k-1}$ could be obtained based on Equation (26). In addition, the initial values of ${\hat{x}}_{k|k},P_{k|k}$ are set as ${\hat{x}}_{0|0}=0,P_{0|0}=0$ based on $x_{0}=0$ in Equation (1).

Iterating the above KF process step by step, the posterior distribution of $x_{k}$ could be solved analytically. Based on the implicit state updating results, the RUL estimation could be conducted.

## 5. Case Study

To verify the feasibility of the proposed method, a numerical simulation and a practical example of the high-voltage pulse capacitor are provided. For performance evaluation, the prediction results of the proposed method are compared with the two-phase nonlinear degradation model considering unit-to-unit variability (Lin’s method) [33], the two-phase linear-nonlinear degradation model with measurement errors (Chai’s method) [37], and the traditional single-phase nonlinear degradation model with three-source variability (Zheng’s method) [25]. The implementation details of the experiment are as follows.

### 5.1. Numerical Example

**Figure 1:**
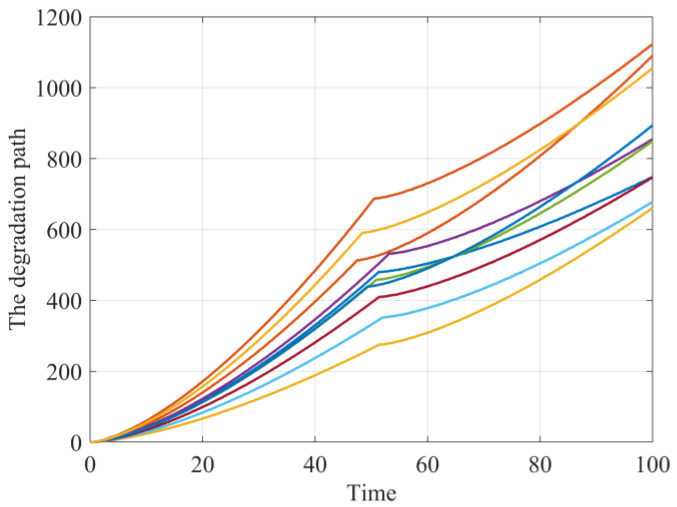
The simulated degradation paths.

In this subsection, we focus on validating the effectiveness of our method for parameter identification and RUL estimation. For illustration purposes, the following power model is applied to define the nonlinear integral term in Equation (1), i.e., $\int_{0}^{t}\mu_{1}(\rho ;\vartheta_{1})d\rho =t^{b_{1}},\int_{\tau }^{t}\mu_{2}(\rho -\tau ;\vartheta_{2})d\rho ={\left(t-\tau \right)}^{b_{2}}$. This kind of power function has been widely used in existing literature [22]. Similar to the work of Feng et al. [20] we adopt the state space model to generate the simulation data and the parameters are given as: $\lambda_{1p}=1.2,\;\sigma_{1p}=0.2,\;b_{1}=1.5,\;\sigma_{1}=0.05,\;\sigma_{1\varepsilon }=0.3,\;\lambda_{2p}=1.5,\;\sigma_{2p}=0.25,\;b_{2}=1.4,\;\sigma_{2}=0.08,\;\sigma_{2\varepsilon }=0.4$ and the distribution parameters of the random changing time, i.e., $\mu_{\tau }=50,\;\sigma_{\tau }=2$. On this basis, we generate 100 sets of degradation trajectories with random changing time, drift parameters, and measurement errors. Figure 1 shows several typical degradation paths of the sample, and it is observable that the degradation paths exhibit obvious two-phase nonlinear deteriorating patterns.

**Figure 2:**
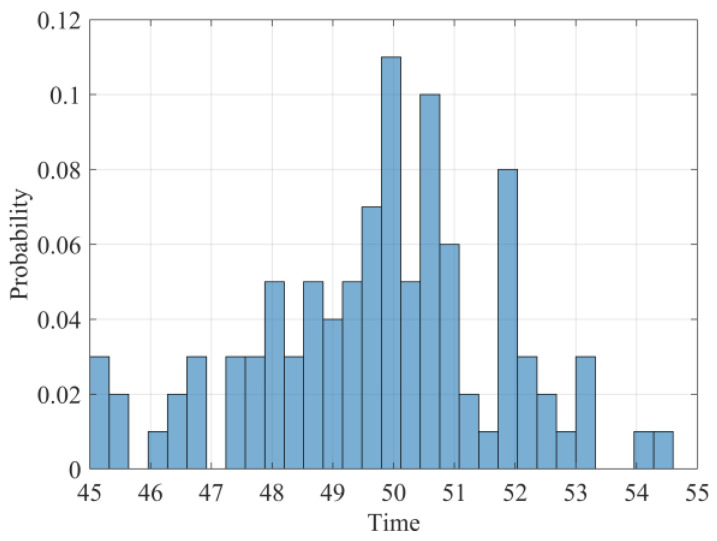
The estimated changing time of 100 degradation paths.

Next, the unknown model parameters are identified based on the changing time detection and parameter estimation methods proposed in Section 4. The parameter estimation results with different sample sizes are detailed in Table 1. It can be found from Table 1 that the obtained results could gradually approach the true values as the size of the degradation paths is increased. In addition, the histogram of the detected changing time based on 100 sample paths is presented in Figure 2 and it manifests that the estimated changing points are concentrated around t=50, which are very close to the true value of $\mu_{\tau }$. Thus, the effectiveness of the offline parameter identification method is demonstrated.

**Figure 3:**
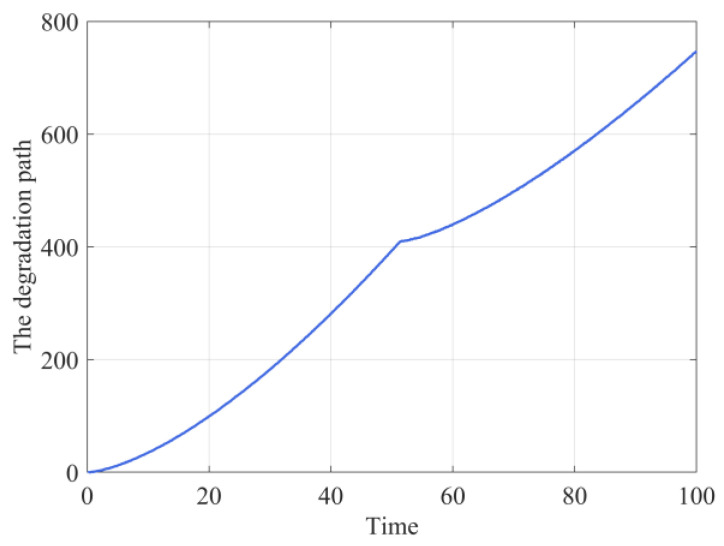
The online degradation path.

For the online parameter and the underlying degradation state updating, we chose one specific degradation path from the above-presented 100 degradation trajectories and the true drift parameters are $\lambda_{1}=1.1133,\lambda_{2}=1.4690$, and τ=51.4 as shown in Figure 3.

**Figure 4:**
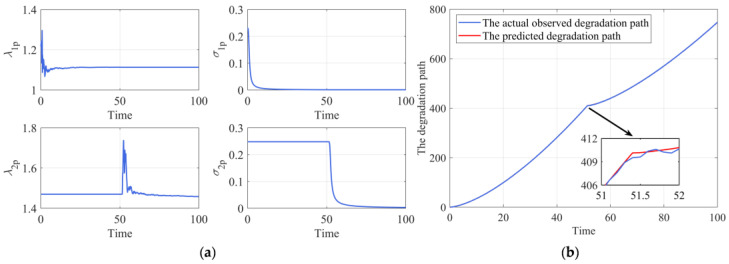
The online updating process. (**a**) The parameter updating. (**b**) The underlying degradation state updating.

Then the changing point detection procedure is applied for the online path, and the estimated changing time is 51.3, which is very close to the true value. Meanwhile, the parameter estimation results of n=10 size in Table 1 are used as the prior information, when newly observed data are coming, the hyper-parameters of the drift coefficients and the underlying degradation state could be updated via the methods proposed in Section 4.3. Figure 4a shows the parameter updating process, it could be observed that the parameter updating curves could gradually approach the true values. Furthermore, it is clear from Figure 4a that as the observations accumulate, the values of $\sigma_{1p}$ and $\sigma_{2p}$ are gradually decreasing, which means the uncertainty of estimation is reduced. In addition, Figure 4b presents the comparison of the predicted underlying degradation path and the observed degradation path. It is clear from Figure 4b that our degradation state updating method could track the actual observed degradation state well.

**Figure 5:**
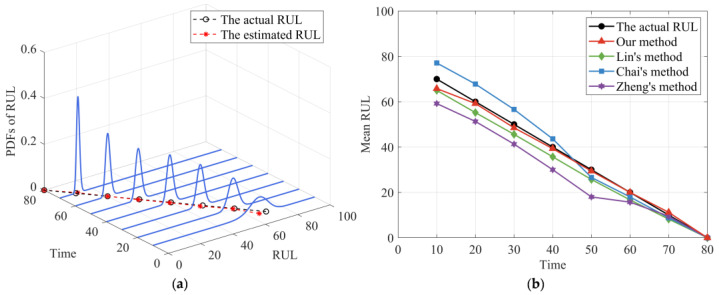
The RUL prediction results. (**a**) PDFs of the RUL. (**b**) The mean RUL of the four methods.

Based on the results of the parameter identification process, the PDFs of RUL estimation at different observed time points could be obtained, as shown in Figure 5a. It is worth noting that the preset failure threshold w=570, thus the actual lifetime is T=80. It can be observed from Figure 5a that the PDFs of RUL estimated by our model are closely distributed around the actual RUL values, verifying that our proposed method could effectively estimate the RUL of the two-phase nonlinear degradation process. For comparative purposes, we further obtained the mean RUL prediction results of our method, Lin’s method [33], Chai’s method [37], and Zheng’s method [25], as shown in Figure 5b. It is observable from Figure 5b that the mean predicted RUL of our method is more accurate compared to the other three methods. Additionally, the mean RUL prediction curve of Lin’s method [33] is relatively stable due to the degradation model they constructed considering two-phase nonlinearity. However, since they ignored the measurement variability, the deviations of their mean RUL prediction curve from the actual values are bigger than our method. Furthermore, the mean RUL curve of Chai’s method [37] in the first phase has large bias, but it gradually becomes accurate in the second phase. Because Chai’s method [37] only consider the nonlinearity of the second phase. In addition, the deviations between the mean RUL prediction curve of Zheng’s method [25] and the actual RUL are the largest among the four methods in the first phase, especially near the changing point where the deviation is more pronounced. It is mainly because the degradation model they constructed is single-phase, which is insufficient to describe the two-phase nonlinear degradation process effectively. It is interesting to note that after the changing point has appeared, the deviations of Zheng’s method [25] gradually decrease as the observations accumulate. The reason for this phenomenon is that the degradation trajectory is equivalent to a single-phase nonlinear degradation process after the changing point appears, thus, Zheng’s method [25] that considers three-source variability simultaneously could effectively fit the degradation process.

For performance evaluation, two metrics are employed to evaluate the performance of RUL prediction among different methods, including the mean square error (MSE) [22] and the absolute error (AE) [37]. The MSE at each observation point could be defined as follows,

(29) $$MSE_{k}=\int_{0}^{+\infty }{\left({\tilde{l}}_{k}-l_{k}\right)}^{2}f_{L_{k}|Y_{1:k}}(l_{k}|Y_{1:k})dl_{k}\;$$

where ${\tilde{l}}_{k}$ denotes the actual RUL at $t_{k}$, and $f_{L_{k}|Y_{1:k}}(l_{k}|Y_{1:k})$ is the corresponding conditional PDF of the RUL estimated by Theorem 2 in Section 3.2.

The second metric is the AE of actual RUL and the estimated RUL at each observation point, which could be represented by

(30) $$AE_{k}=\left|{\tilde{l}}_{k}-{\hat{l}}_{k}\right|\;$$

where ${\hat{l}}_{k}$ denotes the estimated RUL at $t_{k}$.

It is noted that in both criteria, the smallest value of MSE and AE corresponds to the best RUL prediction result.

**Figure 6:**
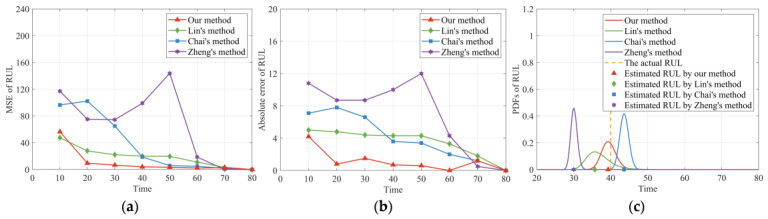
Performance evaluation of the RUL prediction. (**a**) MSE of the estimated RUL. (**b**) AE of the estimated RUL. (**c**) PDFs of the estimated RUL at time 40.

The following Figure 6 presents the comparison results of the four methods. It can be seen from Figure 6a that our method provides smaller MSE values than the other three methods. Additionally, the AE values of our method in Figure 6b are also the smallest of the four models. The comparison results of these two criteria indicate that our RUL prediction method could effectively improve the prediction accuracy of RUL. Moreover, it can be observed that the MSE and AE values of Zheng’s method [25] are the largest of the four methods near the changing point. Because the single-phase nonlinear degradation model constructed by Zheng’s method [25] did not consider the impact of the random degradation state at the changing point on RUL estimation, and cannot estimate the RUL of the two-phase nonlinear degradation process well. In addition, Figure 6c compares the PDFs of the RUL distributions derived from our model with the other three methods at time 40. It is clear from Figure 6c that our method can cover the actual RUL well, which reflects the superiority of our method. Moreover, it can also be observed from Figure 6c that the PDF curve of Zheng’s method [25] cannot cover the true value of RUL well, revealing that the bias of RUL predicted by Zheng’s method [25] is very large in the first phase, which is consistent with the previous results of MSE and AE. It is noteworthy that the units are omitted in Figure 1, Figure 2, Figure 3, Figure 4, Figure 5 and Figure 6 since the degradation data are generated via simulation.

To quantitatively compare the RUL prediction performance of our method with the other methods, we further employ three evaluation metrics. The first metric is the total MSE (TMSE) [19], which is defined as the sum of the MSE at each observation point over the whole life cycle. Videlicet, if there are m observations, the TMSE could be formulated as,

(31) $$TMSE=\sum_{k=1}^{m}MSE_{k}$$

The second metric is the mean absolute error (MAE) [41], which measures the average absolute deviation between the predicted value and the actual value. The MAE could be represented as,

(32) $$MAE=\frac{1}{m}\sum_{k=1}^{m}AE_{k}$$

where $AE_{k}$ is defined in Equation (30).

The third metric is the cumulative relative accuracy (CRA) [27]., which evaluates the relative prediction accuracy of the RUL over time, and could be defined as,

(33) $$CRA=\frac{1}{m}\sum_{k=1}^{m}\left(1-\frac{AE_{k}}{{\tilde{l}}_{k}}\right)$$

For these three quantitative metrics, the smallest value of TMSE and MAE corresponds to the best RUL prediction result, in contrast, a higher CRA value that approaches to 1 indicates higher RUL prediction accuracy. Then, the subsequent Table 2 shows the comparison results of the estimated RUL based on the three metrics mentioned above. It can be found from Table 2 that compared with the other three methods, our method has smaller TMSE and MAE values, whereas a bigger CRA value, which indicates that our method has higher accuracy in RUL prediction.

Overall, the numerical simulation results demonstrate that the proposed prediction RUL method considering three-source variability and two-phase nonlinearity is effective, and could improve the estimation accuracy.

### 5.2. Practical Application

In this subsection, the data of high-voltage pulse capacitors are utilized to illustrate our approach [39]. High-voltage-pulse capacitors can be utilized for electrical energy storing and releasing, which are often encountered in pulse lasers, radar, and particle accelerators [34]. It is well known that capacitors and batteries can form hybrid energy storage systems, which are commonly used as energy sources for hybrid electric vehicles [42,43,44]. However, the unit-to-unit variability mentioned in this work is defined as the heterogeneity between different units in the same batch of identical devices [33,34]. Therefore, if degradation modeling with three-source variability is based on the degradation data of different types of devices, such as capacitors and batteries, the basic premise for the unit-to-unit variability is not satisfied. In addition, if the research object is a system composed of batteries and capacitors, it is another topic of RUL prediction, namely, the RUL estimation for multi-component systems. Currently, our work mainly focuses on the RUL prediction with three-source variability of different devices from the same batch, thus, only the capacitor degradation data is considered in this research.

**Figure 7:**
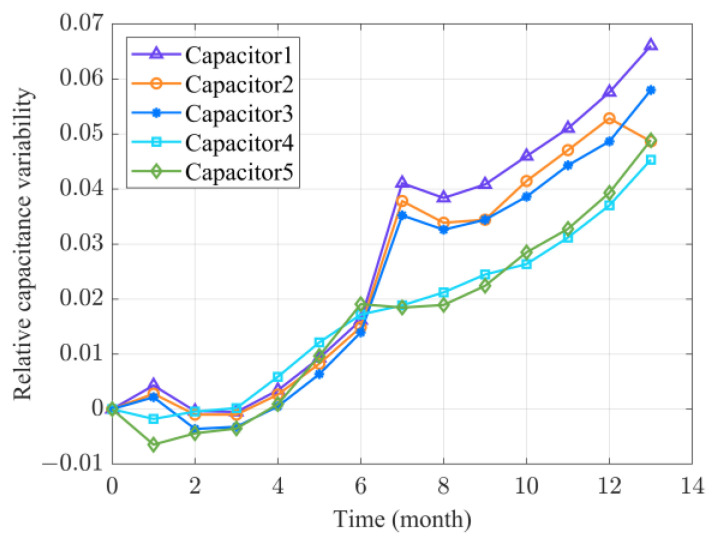
Relative capacitance variability of the capacitors with time.

According to the usage scenario, the high-voltage pulse capacitors have a short service time and a long storage time. Hence, the storage performance is a primary factor that influences the reliability of such capacitors. Capacitance can be used as a health indicator (HI) to evaluate the RUL of the capacitors; Figure 7 shows the degradation test data of five high-voltage pulse capacitors under the storage condition. The capacitance was observed every month and the relative capacitance variability was selected as the HI. Reference [34] revealed that the degradation paths of such capacitors could be modeled based on the two-phase nonlinear method. In addition, Reference [39] mentioned that the capacitance degradation path is affected by temperature and humidity, thus, measurement errors are inevitable. Therefore, it is appropriate to use the degradation data of these capacitors to verify our proposed two-phase nonlinear degradation model.

Based on the degradation data of Capacitors 2–5, the offline parameter estimation results are obtained through the parameter identification method proposed in Section 4, and are listed in Table 3. Through the estimated values of $b_{1},b_{2}$ and $\sigma_{1\varepsilon },\sigma_{2\varepsilon }$ in Table 3, it can be found that nonlinearity and measurement errors exist in the degradation process of capacitors. It is noticeable that the results of Table 3 are treated as the prior information.

**Figure 8:**
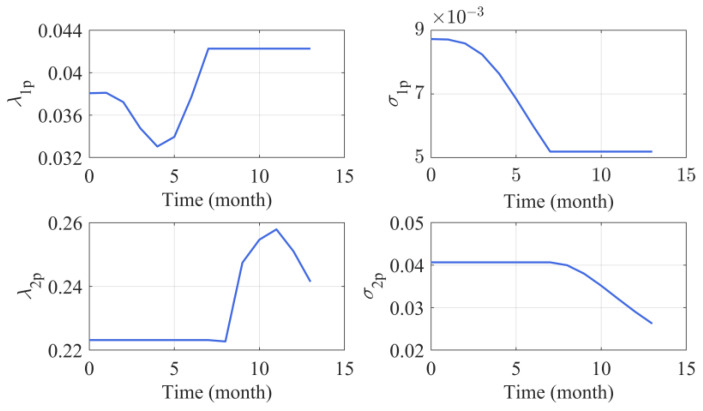
Online parameter updating processes of Capacitor 1.

For online implementation processes, the degradation data of Capacitors 1 is selected to illustrate the parameter updating and RUL prediction processes. Figure 8 presents the parameter updating results, and it can be found that the updated values of $\lambda_{1p},\lambda_{2p}$ are bigger than the offline values in Table 3. The reason is that the degradation path of Capacitors 1 is steeper than the mean paths of Capacitors 2–5, which can be seen in Figure 7.

**Figure 9:**
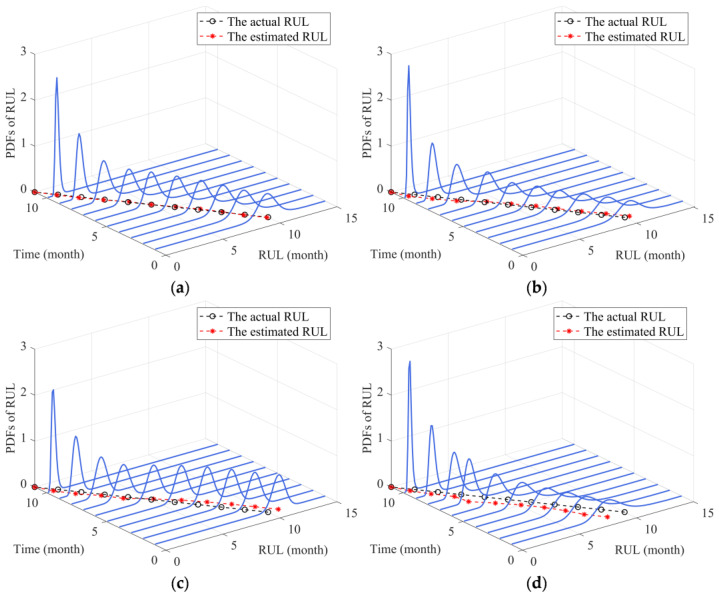
PDFs of RUL prediction for Capacitor 1. (**a**) Our method. (**b**) Lin’s method. (**c**) Chai’ method. (**d**) Zheng’s method.

According to the literature [34,39], the failure threshold of capacitor is set as w=5%. Then, based on the results of model updates, the PDFs of RUL could be obtained as shown in Figure 9a. It is observable from Figure 9a that the estimated values of RUL are almost consistent with the actual values, indicating that our method can effectively estimate the RUL of capacitor degradation data. For comparative purposes, we further presented the estimated RUL of Lin’s method [33], Chai’s method [37], and Zheng’s method [25] as shown graphically in Figure 9b–d, respectively. It can be observed from Figure 9 that in both phases, the PDF curves of the proposed method are sharper compared to Lin’s method [33] and Zheng’s method [25]. Additionally, although our PDF curves are similar in steepness to Chai’s method [37], the PDF curves of our method are more compact around the actual RUL and the estimated RUL values are closer to the actual values compared to the other three methods. Thus, from the overall RUL prediction results, our method that considers three-source variability and two-phase nonlinearity has higher RUL prediction accuracy compared to the other three methods.

To evaluate the performance of RUL prediction, three metrics, including MSE, AE, and relative error (RE) [19], are adopted for performance evaluation. Among them, the RE of the estimated RUL at $t_{k}$ can be defined as,

(34) $$RE_{k}=\frac{\left|{\tilde{l}}_{k}-{\hat{l}}_{k}\right|}{\tilde{l}}\;$$

where ${\tilde{l}}_{k}$ and ${\hat{l}}_{k}$ denotes the estimated and the actual RUL at $t_{k}$, respectively.

**Figure 10:**
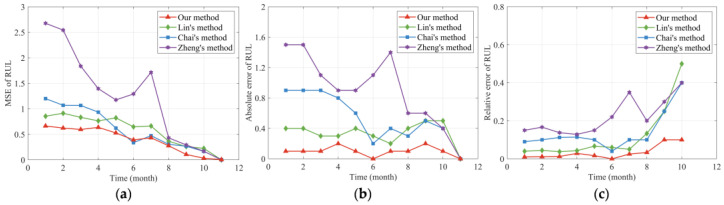
Performance evaluation based on capacitance degradation data of Capacitor 1. (**a**) MSE of the predicted RUL. (**b**) AE of the predicted RUL. (**c**) RE of the predicted RUL.

**Figure 11:**
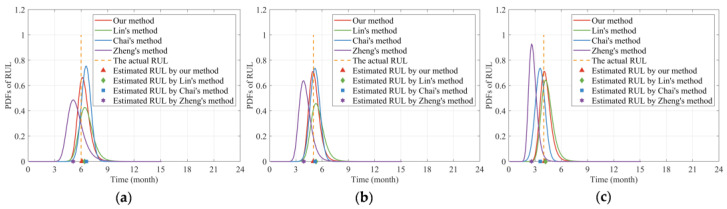
Comparison of the estimated RUL at different months. (**a**) PDFs of the estimated RUL at the fifth month. (**b**) PDFs of the estimated RUL at the sixth month. (**c**) PDFs of the estimated RUL at the seventh month.

Then, we calculated the MSE, AE, and RE of the predicted RUL, as shown in Figure 10. It can be found from Figure 10a that the MES values of our method maintain a relatively low level compared to the other three methods. Additionally, it can be observed from Figure 10b,c that the AE as well as the RE results of the proposed method are smaller than the other three methods, indicating the higher accuracy of our proposed method. Furthermore, it can be seen from Figure 10c that the RE curves of each method gradually increase in the later stage of the degradation process. The main reason is that as time accumulates, the actual value of RUL becomes smaller and smaller. Although the AE value is gradually decreasing over time, its proportion in the actual RUL value is larger than that in the early stage of the degradation process, thus, resulting in an increase trend in the later stage. In addition, it can be seen from Figure 10a that the MSE value of Chai’s method [37] is slightly better than the MES value of our method. To investigate the reasons for this phenomenon, we further obtained the PDFs of the RUL estimate in the fifth, sixth, and seventh adjacent months, as shown in Figure 11.

It can be found from Figure 11 that the PDF curves of our method at the three time points can cover the actual values of RUL well, and are more compact around the actual RUL, respectively. Moreover, the estimated RUL values of our method at different time points are closer to the actual values than the other three methods.

The key issue lies in the PDF curve of the estimated RUL at the sixth month in Figure 11b. As can be seen from Figure 11b, the deviation between our estimated RUL and the actual value is only slightly smaller than that of Chai’s method [37]. However, the PDF curve of Chai’s method [37] is sharper than ours. According to the definition of MSE in Equation (29), the MSE value is jointly affected by the deviation between the estimated value of RUL and the actual value, as well as the corresponding PDF function. The MSE value will be smaller when the PDF of RUL is closely distributed around the actual RUL value [34]. Therefore, the MSE value of Chai’s method [37] is slightly smaller than ours in the sixth month. In fact, this is acceptable. Generally, as can be seen in Figure 10a, the MSE curve of our proposed method maintains a relatively low level compared to Chai’s method [37]. Therefore, the MSE result in the sixth month does not affect the conclusion that our RUL prediction accuracy is higher than Chai’s method [37].

To quantitatively compare the predicted results based on the capacitor degradation data, we further calculated the TMSE, MAE, and CRA values of RUL estimation, as detailed in Table 4. It is observable from Table 4 that the TMSE of our method is the smallest. Thus, although the MSE value of Chai’s method [37] in Figure 10a is smaller than ours in the sixth month, however, in general, the TMSE of our method is still better than Chai’s method [37]. In addition, among the four methods, our method has the smallest MAE value and the largest CRA value, because we simultaneously consider the two-phase nonlinearity and the three-source variability of the degradation process. The results of the above quantitative metrics indicate that our RUL prediction method has higher accuracy compared to the other three methods, which verifies the effectiveness and superiority of our method.

In summary, the experimental results show that compared to the other three methods, the MSE, AE, and RE curves of our method maintain a relatively low level, and the RUL prediction results of our method have smaller TMSE, MAE values, whereas larger CRA values. Based on the above results, it can be found that our work can obtain competitive results, which indicates that the accuracy of RUL prediction is better than the other three methods, thus, verifying the feasibility and effectiveness of the proposed method in practical application. Furthermore, for two-phase nonlinear degradation devices, it is necessary to consider the nonlinearity and the three-source variability simultaneously to boost the performance of RUL prediction.

## 6. Conclusions

In this study, a two-phase nonlinear Wiener process-based degradation model subject to the three-source variability is formulated for RUL prediction. Based on the FPT concept, the approximate analytical form of the RUL is obtained considering the three-source variability and the random degradation state at the changing point simultaneously. To incorporate the historical observations of the devices within the same batch, the MLE method is adopted to estimate the unknown model parameters. By combining the Bayesian rule and KF algorithm, the drift coefficients and the underlying degradation state are adaptively updated in real-time with newly observed data. Finally, the effectiveness of the proposed method is verified via numerical and practical examples. The quantitative comparison results of MSE, AE, RE, TMSE, MAE, and CRA reveal that the two-phase nonlinear degradation model with three-source variability is more accurate than the existing methods that only partially consider the two-phase nonlinearity and three-source variability. Therefore, in RUL prediction for the two-phase nonlinear degradation devices, the impact of these uncertainties on degradation modeling should be considered simultaneously to improve the accuracy of RUL prediction. However, there are several directions that are worth further study. First, the measurement error is assumed as Gaussian, which may be inadequate when there are outliers in the degradation observations. In this case, the non-Gaussian measurement errors need to be considered, such as t-distribution. Second, the proposed model is formulated under the assumption of the progressive degradation process. However, shocks are often encountered in practice. Therefore, determining how to construct the two-phase nonlinear degradation model considering the interaction between the internal degradation mechanism and external shocks needs to be explored. Third, at present, our research only focuses on the RUL prediction for single-component systems, without considering the impact of correlation between different components on RUL prediction. Therefore, the degradation modeling and RUL prediction of multi-component systems (such as hybrid energy systems consisting of capacitors and batteries) with three-source variability is also a valuable research direction in the future.

## Figures and Tables

**Table 1:** Parameter estimation values with different sample sizes.

Size	$\lambda_{1p}$	$\sigma_{1p}$	$\sigma_{1}$	$\sigma_{1\varepsilon }$	$b_{1}$	$\lambda_{2p}$	$\sigma_{2p}$	$\sigma_{2}$	$\sigma_{2\varepsilon }$	$b_{2}$	$\mu_{\tau }$	$\sigma_{\tau }$
n=10	1.3279	0.3370	0.0450	0.2963	1.4994	1.6560	0.2851	0.0972	0.3967	1.4027	50.5800	1.6302
n=50	1.2559	0.2500	0.0518	0.2983	1.5002	1.4794	0.2557	0.0973	0.4000	1.4016	49.6180	2.1199
n=100	1.2243	0.2311	0.0505	0.2995	1.5001	1.4865	0.2482	0.1041	0.4024	1.4017	49.7400	1.9923
True value	1.2	0.2	0.05	0.3	1.5	1.5	0.25	0.08	0.4	1.4	50	2

**Table 2:** Comparison results of RUL prediction based on TMSE, MAE, and CRA.

Metric	TMSE	MAE	CRA
Our method	1.2030 × 10^4^	0.8634	0.9653
Lin’s method [33]	2.3221 × 10^4^	3.7415	0.8795
Chai’s method [37]	3.1273 × 10^4^	3.9529	0.8976
Zheng’s method [25]	6.9231 × 10^4^	8.3726	0.8037

**Table 3:** The parameter estimation results of the capacitance degradation data.

Variable	$\lambda_{1p}$	$\sigma_{1p}$	$\sigma_{1}$	$\sigma_{1\varepsilon }$	$b_{1}$	$\lambda_{2p}$	$\sigma_{2p}$	$\sigma_{2}$	$\sigma_{2\varepsilon }$	$b_{2}$	$\mu_{\tau }$	$\sigma_{\tau }$
value	0.0381	0.0087	0.1614	0.0497	2.0800	0.2232	0.0407	0.2274	0.0344	1.5217	6.2500	0.8292

**Table 4:** Comparison results of RUL prediction based on TMSE, MAE, and CRA for Capacitor 1 degradation data.

Metric	TMSE	MAE	CRA
Our method	4.9349	0.1091	0.9685
Lin’s method [33]	7.0592	0.3637	0.8862
Chai’s method [37]	7.5904	0.6182	0.8647
Zheng’s method [25]	16.2556	1.0455	0.7873

## Data Availability

The data presented in this study are openly available in Ref. [39].

## Nomenclature

The following abbreviations and notations are used in this manuscript:

Abbreviations	
PHM	Prognostics and health management
RUL	Remaining useful life
PDF	Probability density function
CDF	Cumulative distribution function
CM	Condition monitoring
KF	Kalman filtering
EM	Expectation maximization
ELM	Extreme learning machine
FPT	First passage time
MSE	Mean square error
AE	Absolute error
HI	health indicator
Notation	
X(t)	Actual degradation state at time t
$x_{0}$	Initial value
τ	Time of the changing point
$x_{\tau }$	Degradation state at the changing time
$\lambda_{1}$	Drift coefficient of the first phase
$\lambda_{2}$	Drift coefficient of the second phase
$\sigma_{1}$	Diffusion coefficients of the first phase
$\sigma_{2}$	Diffusion coefficients of the second phase
$\mu_{1}(\rho ;\vartheta_{1})$	Nonlinear function of the first phase
$\mu_{2}(\rho -\tau ;\vartheta_{2})$	Nonlinear function of the second phase
$\vartheta_{1}$	$\mathrm{Nonlinear}\;\mathrm{parameter}\;\mathrm{in}\;\mu_{1}(\rho ;\vartheta_{1})$
$\vartheta_{2}$	$\mathrm{Nonlinear}\;\mathrm{parameter}\;\mathrm{in}\;\mu_{2}(\rho -\tau ;\vartheta_{2})$
B(t)	Standard Brown motion
$\lambda_{1p},\sigma_{1p}$	$\mathrm{Mean}\;\mathrm{and}\;\mathrm{standard}\;\mathrm{deviation}\;\mathrm{of}\;\lambda_{1}$
$\lambda_{2p},\sigma_{2p}$	$\mathrm{Mean}\;\mathrm{and}\;\mathrm{standard}\;\mathrm{deviation}\;\mathrm{of}\;\lambda_{2}$
Y(t)	Measurement process
$\varepsilon_{1},\varepsilon_{2}$	Measurement errors of each phase
$\sigma_{1\varepsilon },\sigma_{2\varepsilon }$	$\mathrm{Standard}\;\mathrm{deviation}\;\mathrm{of}\;\varepsilon_{1}$$\;\mathrm{and}\;\varepsilon_{2}$
w	Failure threshold
T	Lifetime
$L_{k}$	$\mathrm{RUL}\;\mathrm{at}\;\mathrm{the}\;\mathrm{current}\;\mathrm{time}\;t_{k}$
$Y_{1:k}$	$\mathrm{Observations}\;\mathrm{up}\;\mathrm{to}\;t_{k}$
$f_{T}(\cdot )$	PDF of the lifetime
$f_{L}(\cdot )$	PDF of the RUL
Φ	CDF of the standard normal distribution
ϕ	PDF of the standard normal distribution
$h_{\tau }(x_{\tau })$	Transition probability density function
$x_{k}$	$\mathrm{Actual}\;\mathrm{degradation}\;\mathrm{state}\;\mathrm{at}\;\mathrm{the}\;\mathrm{current}\;\mathrm{time}\;t_{k}$
${\hat{x}}_{k|k},P_{k|k}$	$\mathrm{Mean}\;\mathrm{and}\;\mathrm{variance}\;\mathrm{of}\;x_{k}$
Θ	Unknown model parameter vector
N	Number of the tested devices from the same batch
$Y_{1:N}$	Historical data of N devices from the same batch
$X_{1:N}$	Actual degradation state data of N devices from the same batch
$Y_{n}$	Observations of the *n*-*th* device
$\Delta y_{n,j}$	$\mathrm{Measured}\;\mathrm{increment}\;\mathrm{at}\;\mathrm{time}\;t_{j}$of the *n*-*th* device
$\Delta Y_{n}$	Measured increment vector
$m_{n}$	Available number of the observations for the *n*-*th* device
$\tau_{n}$	Changing time of the *n*-*th* device
${\tilde{\tau }}_{n}$	Changing point location of the *n*-*th* device
$\mu_{\tau },\sigma_{\tau }$	Mean and standard deviation of τ
$Y_{1:k}$	$\mathrm{Degradation}\;\mathrm{observations}\;\mathrm{at}\;\mathrm{the}\;\mathrm{current}\;\mathrm{time}\;t_{k}$
$X_{1:k}$	$\mathrm{actual}\;\mathrm{degradation}\;\mathrm{states}\;\mathrm{at}\;\mathrm{the}\;\mathrm{current}\;\mathrm{time}\;t_{k}$

## Appendix A. Proof of Theorem 1

**Proof.** To obtain the RUL estimation result considering the unit-to-unit variability and the random degradation state at the changing point, the PDF of the lifetime should be derived first, which is given by Ref. [33]. For the two-phase nonlinear degradation model proposed in Equation (1), if the drift coefficient in each phase is a random variable and follows a normal distribution, i.e., $\lambda_{1}\sim N(\lambda_{1p},\sigma_{1p}^{2}),\lambda_{2}\sim N(\lambda_{2p},\sigma_{2p}^{2})$, the PDF of the lifetime T considering the unit-to-unit variability and the random degradation state at the changing point could be expressed as follows,

(A1) $$f_{T}(t)≅\left\{\begin{array}{ll} \frac{\left[w-\left(\int_{0}^{t}\mu_{1}(\rho ;\vartheta_{1})d\rho -t\mu_{1}(t;\vartheta_{1})\right)\times \frac{\sigma_{1p}^{2}w\int_{0}^{t}\mu_{1}(\rho ;\vartheta_{1})d\rho +\lambda_{1p}\sigma_{1}^{2}t}{{\left(\int_{0}^{t}\mu_{1}(\rho ;\vartheta_{1})d\rho \right)}^{2}\sigma_{1p}^{2}+\sigma_{1}^{2}t}\right]}{\sqrt{2\pi t^{2}\left({\left(\int_{0}^{t}\mu_{1}(\rho ;\vartheta_{1})d\rho \right)}^{2}\sigma_{1p}^{2}+\sigma_{1}^{2}t\right)}}\exp\left[-\frac{{\left(w-\lambda_{1p}\int_{0}^{t}\mu_{1}(\rho ;\vartheta_{1})d\rho \right)}^{2}}{2\left({\left(\int_{0}^{t}\mu_{1}(\rho ;\vartheta_{1})d\rho \right)}^{2}\sigma_{1p}^{2}+\sigma_{1}^{2}t\right)}\right], & 0<t\leq \tau \\ Q-R, & t>\tau \end{array}\right.\;$$

where $Q=Q_{1}-Q_{2}$, $R=R_{1}-R_{2}$, and

$$\begin{array}{l} Q_{1}=\sqrt{\frac{r_{a1}^{2}}{\sqrt{2\pi {\left(t-\tau \right)}^{2}(\sigma_{\alpha 1}^{2}+\sigma_{\beta 1}^{2})}}}\exp\left[-\frac{{\left(\lambda_{\alpha 1}-\lambda_{\beta 1}\right)}^{2}}{2(\sigma_{\alpha 1}^{2}+\sigma_{\beta 1}^{2})}\right]\\ \times \left\{\frac{\lambda_{\beta 1}\sigma_{\alpha 1}^{2}+\lambda_{\alpha 1}\sigma_{\beta 1}^{2}}{\sigma_{\alpha 1}^{2}+\sigma_{\beta 1}^{2}}\times \Phi \left(\frac{\lambda_{\beta 1}\sigma_{\alpha 1}^{2}+\lambda_{\alpha 1}\sigma_{\beta 1}^{2}}{\sqrt{\sigma_{\alpha 1}^{2}\sigma_{\beta 1}^{2}(\sigma_{\alpha 1}^{2}+\sigma_{\beta 1}^{2})}}\right)+\sqrt{\frac{\sigma_{\alpha 1}^{2}\sigma_{\beta 1}^{2}}{\sigma_{\alpha 1}^{2}+\sigma_{\beta 1}^{2}}}\times \phi \left(\frac{\lambda_{\beta 1}\sigma_{\alpha 1}^{2}+\lambda_{\alpha 1}\sigma_{\beta 1}^{2}}{\sqrt{\sigma_{\alpha 1}^{2}\sigma_{\beta 1}^{2}(\sigma_{\alpha 1}^{2}+\sigma_{\beta 1}^{2})}}\right)\right\}\\ Q_{2}=\sqrt{\frac{r_{b1}^{2}}{2\pi {\left(t-\tau \right)}^{2}(\sigma_{\alpha 1}^{2}+\sigma_{\beta 1}^{2})}}\exp\left[-\frac{{\left(\lambda_{\alpha 1}-\lambda_{\beta 1}\right)}^{2}}{2(\sigma_{\alpha 1}^{2}+\sigma_{\beta 1}^{2})}\right]\times \left\{1-\Phi \left(-\frac{\lambda_{\beta 1}\sigma_{\alpha 1}^{2}+\lambda_{\alpha 1}\sigma_{\beta 1}^{2}}{\sqrt{\sigma_{\alpha 1}^{2}\sigma_{\beta 1}^{2}(\sigma_{\alpha 1}^{2}+\sigma_{\beta 1}^{2})}}\right)\right\}\\ R_{1}=I_{1}\times \sqrt{\frac{r_{a1}^{2}}{2\pi {\left(t-\tau \right)}^{2}(\sigma_{\alpha 1}^{2}+\sigma_{\beta 1}^{2})}}\exp\left[-\frac{{\left(\lambda_{\alpha 1}-\lambda_{\gamma 1}\right)}^{2}}{2(\sigma_{\alpha 1}^{2}+\sigma_{\beta 1}^{2})}\right]\\ \times \left\{\frac{\lambda_{\gamma 1}\sigma_{\alpha 1}^{2}+\lambda_{\alpha 1}\sigma_{\beta 1}^{2}}{\sigma_{\alpha 1}^{2}+\sigma_{\beta 1}^{2}}\times \Phi \left(\frac{\lambda_{\gamma 1}\sigma_{\alpha 1}^{2}+\lambda_{\alpha 1}\sigma_{\beta 1}^{2}}{\sqrt{\sigma_{\alpha 1}^{2}\sigma_{\beta 1}^{2}(\sigma_{\alpha 1}^{2}+\sigma_{\beta 1}^{2})}}\right)+\sqrt{\frac{\sigma_{\alpha 1}^{2}\sigma_{\beta 1}^{2}}{\sigma_{\alpha 1}^{2}+\sigma_{\beta 1}^{2}}}\times \phi \left(\frac{\lambda_{\gamma 1}\sigma_{\alpha 1}^{2}+\lambda_{\alpha 1}\sigma_{\beta 1}^{2}}{\sqrt{\sigma_{\alpha 1}^{2}\sigma_{\beta 1}^{2}(\sigma_{\alpha 1}^{2}+\sigma_{\beta 1}^{2})}}\right)\right\}\\ R_{2}=I_{1}\times \sqrt{\frac{r_{b1}^{2}}{2\pi {\left(t-\tau \right)}^{2}(\sigma_{\alpha 1}^{2}+\sigma_{\beta 1}^{2})}}\exp\left[-\frac{{\left(\lambda_{\alpha 1}-\lambda_{\gamma 1}\right)}^{2}}{2(\sigma_{\alpha 1}^{2}+\sigma_{\beta 1}^{2})}\right]\times \left\{1-\Phi \left(-\frac{\lambda_{\gamma 1}\sigma_{\alpha 1}^{2}+\lambda_{\alpha 1}\sigma_{\beta 1}^{2}}{\sqrt{\sigma_{\alpha 1}^{2}\sigma_{\beta 1}^{2}(\sigma_{\alpha 1}^{2}+\sigma_{\beta 1}^{2})}}\right)\right\}\\ \lambda_{\alpha 1}=\lambda_{2p}\int_{\tau }^{t}\mu_{2}(\rho -\tau ;\vartheta_{2})d\rho ,\lambda_{\beta 1}=w-\lambda_{1p}\int_{0}^{\tau }\mu_{1}(\rho ;\vartheta_{1})d\rho ,\lambda_{\gamma 1}=-w-\lambda_{1p}\int_{0}^{\tau }\mu_{1}(\rho ;\vartheta_{1})d\rho -\frac{2w\sigma_{1p}^{2}\int_{0}^{\tau }\mu_{1}(\rho ;\vartheta_{1})d\rho }{\sigma_{1}^{2}},\\ \sigma_{\alpha 1}^{2}=\sigma_{2p}^{2}{\left(\int_{\tau }^{t}\mu_{2}(\rho -\tau ;\vartheta_{2})d\rho \right)}^{2}+\sigma_{2}^{2}(t-\tau ),\sigma_{\beta 1}^{2}=\sigma_{1p}^{2}{\left(\int_{0}^{\tau }\mu_{1}(\rho ;\vartheta_{1})d\rho \right)}^{2}+\sigma_{1}^{2}\tau \\ r_{a1}=\frac{(t-\tau )\left(\sigma_{2}^{2}+\sigma_{2p}^{2}\mu_{2}(t-\tau ;\vartheta_{2})\int_{\tau }^{t}\mu_{2}(\rho -\tau ;\vartheta_{2})d\rho \right)}{\sigma_{\alpha 1}^{2}},r_{b1}=\frac{(t-\tau )\sigma_{2}^{2}\left(\lambda_{\alpha 1}-\lambda_{2p}(t-\tau )\mu_{2}(t-\tau ;\vartheta_{2})\right)}{\sigma_{\alpha 1}^{2}}\\ I_{1}=\exp\left(\frac{2\lambda_{1p}w}{\sigma_{1}^{2}}+\frac{2(w^{2}\sigma_{1p}^{4}\int_{0}^{\tau }\mu_{1}(\rho ;\vartheta_{1})d\rho +w^{2}\sigma_{1p}^{2}\sigma_{1}^{2})}{(\sigma_{1}^{2}+\sigma_{1p}^{2}\int_{0}^{\tau }\mu_{1}(\rho ;\vartheta_{1})d\rho )\sigma_{1}^{4}}\right) \end{array}$$

Based on Equation (A1), according to the relationship between the lifetime T and RUL, the PDF of RUL that considers temporal variability, unit-to-unit variability, and the randomness of the degradation state at the changing point could be obtained as follows, Case 1: The current time $t_{k}$ is smaller than the changing time τ (i.e., $t_{k}<\tau$)

(A2) $$f_{L}(l_{k})≅\left\{\begin{array}{ll} \begin{array}{l} \frac{1}{\sqrt{2\pi l_{k}{}^{2}\left({\left(\int_{t_{k}}^{t_{k}+l_{k}}\mu_{1}(\rho ;\vartheta_{1})d\rho \right)}^{2}\sigma_{1p}^{2}+\sigma_{1}^{2}l_{k}\right)}}\times \left[w-x_{k}-\left(\int_{t_{k}}^{t_{k}+l_{k}}\mu_{1}(\rho ;\vartheta_{1})d\rho -l_{k}\mu_{1}(t_{k}+l_{k};\vartheta_{1})\right)\right.\\ \left.\times \frac{(w-x_{k})\sigma_{1p}^{2}\int_{t_{k}}^{t_{k}+l_{k}}\mu_{1}(\rho ;\vartheta_{1})d\rho +\lambda_{1p}\sigma_{1}^{2}l_{k}}{{\left(\int_{t_{k}}^{t_{k}+l_{k}}\mu_{1}(\rho ;\vartheta_{1})d\rho \right)}^{2}\sigma_{1p}^{2}+\sigma_{1}^{2}l_{k}}\right]\times \exp\left[-\frac{{\left(w-x_{k}-\lambda_{1p}\int_{t_{k}}^{t_{k}+l_{k}}\mu_{1}(\rho ;\vartheta_{1})d\rho \right)}^{2}}{2\left({\left(\int_{t_{k}}^{t_{k}+l_{k}}\mu_{1}(\rho ;\vartheta_{1})d\rho \right)}^{2}\sigma_{1p}^{2}+\sigma_{1}^{2}l_{k}\right)}\right], \end{array} & 0<l_{k}+t_{k}\leq \tau \\ S-T, & \tau <l_{k}+t_{k} \end{array}\right.$$

where $S=S_{1}-S_{2},T=T_{1}-T_{2}$, and

$$\begin{array}{l} S_{1}=\sqrt{\frac{r_{a2}^{2}}{2\pi {\left(t_{k}+l_{k}-\tau \right)}^{2}(\sigma_{\alpha 2}^{2}+\sigma_{\beta 2}^{2})}}\exp\left[-\frac{{\left(\lambda_{\alpha 2}-\lambda_{\beta 2}\right)}^{2}}{2(\sigma_{\alpha 2}^{2}+\sigma_{\beta 2}^{2})}\right]\\ \times \left\{\frac{\lambda_{\beta 2}\sigma_{\alpha 2}^{2}+\lambda_{\alpha 2}\sigma_{\beta 2}^{2}}{\sigma_{\alpha 2}^{2}+\sigma_{\beta 2}^{2}}\times \Phi \left(\frac{\lambda_{\beta 2}\sigma_{\alpha 2}^{2}+\lambda_{\alpha 2}\sigma_{\beta 2}^{2}}{\sqrt{\sigma_{\alpha 2}^{2}\sigma_{\beta 2}^{2}(\sigma_{\alpha 2}^{2}+\sigma_{\beta 2}^{2})}}\right)+\sqrt{\frac{\sigma_{\alpha 2}^{2}\sigma_{\beta 2}^{2}}{\sigma_{\alpha 2}^{2}+\sigma_{\beta 2}^{2}}}\times \phi \left(\frac{\lambda_{\beta 2}\sigma_{\alpha 2}^{2}+\lambda_{\alpha 2}\sigma_{\beta 2}^{2}}{\sqrt{\sigma_{\alpha 2}^{2}\sigma_{\beta 2}^{2}(\sigma_{\alpha 2}^{2}+\sigma_{\beta 2}^{2})}}\right)\right\}\\ S_{2}=\sqrt{\frac{r_{b2}^{2}}{2\pi {\left(t_{k}+l_{k}-\tau \right)}^{2}(\sigma_{\alpha 2}^{2}+\sigma_{\beta 2}^{2})}}\exp\left[-\frac{{\left(\lambda_{\alpha 2}-\lambda_{\beta 2}\right)}^{2}}{2(\sigma_{\alpha 2}^{2}+\sigma_{\beta 2}^{2})}\right]\times \left\{1-\Phi \left(-\frac{\lambda_{\beta 2}\sigma_{\alpha 2}^{2}+\lambda_{\alpha 2}\sigma_{\beta 2}^{2}}{\sqrt{\sigma_{\alpha 2}^{2}\sigma_{\beta 2}^{2}(\sigma_{\alpha 2}^{2}+\sigma_{\beta 2}^{2})}}\right)\right\}\\ T_{1}=I_{2}\times \sqrt{\frac{r_{a2}^{2}}{2\pi {\left(t_{k}+l_{k}-\tau \right)}^{2}(\sigma_{\alpha 2}^{2}+\sigma_{\beta 2}^{2})}}\exp\left[-\frac{{\left(\lambda_{\alpha 2}-\lambda_{\gamma 2}\right)}^{2}}{2(\sigma_{\alpha 2}^{2}+\sigma_{\beta 2}^{2})}\right]\\ \times \left\{\frac{\lambda_{\gamma 2}\sigma_{\alpha 2}^{2}+\lambda_{\alpha 2}\sigma_{\beta 2}^{2}}{\sigma_{\alpha 2}^{2}+\sigma_{\beta 2}^{2}}\times \Phi \left(\frac{\lambda_{\gamma 2}\sigma_{\alpha 2}^{2}+\lambda_{\alpha 2}\sigma_{\beta 2}^{2}}{\sqrt{\sigma_{\alpha 2}^{2}\sigma_{\beta 2}^{2}(\sigma_{\alpha 2}^{2}+\sigma_{\beta 2}^{2})}}\right)+\sqrt{\frac{\sigma_{\alpha 2}^{2}\sigma_{\beta 2}^{2}}{\sigma_{\alpha 2}^{2}+\sigma_{\beta 2}^{2}}}\times \phi \left(\frac{\lambda_{\gamma 2}\sigma_{\alpha 2}^{2}+\lambda_{\alpha 2}\sigma_{\beta 2}^{2}}{\sqrt{\sigma_{\alpha 2}^{2}\sigma_{\beta 2}^{2}(\sigma_{\alpha 2}^{2}+\sigma_{\beta 2}^{2})}}\right)\right\}\\ T_{2}=I_{2}\times \sqrt{\frac{r_{b2}^{2}}{2\pi {\left(t_{k}+l_{k}-\tau \right)}^{2}(\sigma_{\alpha 2}^{2}+\sigma_{\beta 2}^{2})}}\exp\left[-\frac{{\left(\lambda_{\alpha 2}-\lambda_{\gamma 2}\right)}^{2}}{2(\sigma_{\alpha 2}^{2}+\sigma_{\beta 2}^{2})}\right]\times \left\{1-\Phi \left(-\frac{\lambda_{\gamma 2}\sigma_{\alpha 2}^{2}+\lambda_{\alpha 2}\sigma_{\beta 2}^{2}}{\sqrt{\sigma_{\alpha 2}^{2}\sigma_{\beta 2}^{2}(\sigma_{\alpha 2}^{2}+\sigma_{\beta 2}^{2})}}\right)\right\}\\ \lambda_{\alpha 2}=\lambda_{2p}\int_{\tau }^{t_{k}+l_{k}}\mu_{2}(\rho -\tau ;\vartheta_{2})d\rho ,\lambda_{\beta 2}=w-x_{k}-\lambda_{1p}\int_{t_{k}}^{\tau }\mu_{1}(\rho ;\vartheta_{1})d\rho ,\\ \lambda_{\gamma 2}=-w+x_{k}-\lambda_{1p}\int_{t_{k}}^{\tau }\mu_{1}(\rho ;\vartheta_{1})d\rho -\frac{2(w-x_{k})\sigma_{1p}^{2}\int_{t_{k}}^{\tau }\mu_{1}(\rho ;\vartheta_{1})d\rho }{\sigma_{1}^{2}},\\ \sigma_{\alpha 2}^{2}=\sigma_{2p}^{2}{\left(\int_{\tau }^{t_{k}+l_{k}}\mu_{2}(\rho -\tau ;\vartheta_{2})d\rho \right)}^{2}+\sigma_{2}^{2}(t_{k}+l_{k}-\tau ),\sigma_{\beta 2}^{2}=\sigma_{1p}^{2}{\left(\int_{t_{k}}^{\tau }\mu_{1}(\rho ;\vartheta_{1})d\rho \right)}^{2}+\sigma_{1}^{2}(\tau -t_{k}),\\ r_{a2}=\frac{(t_{k}+l_{k}-\tau )\left(\sigma_{2}^{2}+\sigma_{2p}^{2}\mu_{2}(t_{k}+l_{k}-\tau ;\vartheta_{2})\int_{\tau }^{t_{k}+l_{k}}\mu_{2}(\rho -\tau ;\vartheta_{2})d\rho \right)}{\sigma_{\alpha 2}^{2}},\\ r_{b2}=\frac{(t_{k}+l_{k}-\tau )\sigma_{2}^{2}\left(\lambda_{a2}-\lambda_{2p}(t_{k}+l_{k}-\tau )\mu_{2}(t_{k}+l_{k}-\tau ;\vartheta_{2})\right)}{\sigma_{\alpha 2}^{2}},\\ I_{2}=\exp\left[\frac{2\lambda_{1p}(w-x_{k})}{\sigma_{1}^{2}}+\frac{2\left({\left(w-x_{k}\right)}^{2}\sigma_{1p}^{4}\int_{t_{k}}^{\tau }\mu_{1}(\rho ;\vartheta_{1})d\rho +{\left(w-x_{k}\right)}^{2}\sigma_{1p}^{2}\sigma_{1}^{2}\right)}{\left(\sigma_{1}^{2}+\sigma_{1p}^{2}\int_{t_{k}}^{\tau }\mu_{1}(\rho ;\vartheta_{1})d\rho \right)\sigma_{1}^{4}}\right] \end{array}$$

Case 2: The current time $t_{k}$ is larger than the changing time τ (i.e., $t_{k}\geq \tau$)

(A3) $$\begin{array}{l} f_{L}(l_{k})≅\frac{1}{\sqrt{2\pi l_{k}{}^{2}\left({\left(\int_{t_{k}}^{t_{k}+l_{k}}\mu_{2}(\rho -\tau ;\vartheta_{2})d\rho \right)}^{2}\sigma_{2p}^{2}+\sigma_{2}^{2}l_{k}\right)}}\times \left[w-x_{k}-\left(\int_{t_{k}}^{t_{k}+l_{k}}\mu_{2}(\rho -\tau ;\vartheta_{2})d\rho -l_{k}\mu_{2}(t_{k}+l_{k}-\tau ;\vartheta_{2})\right)\right.\\ \left.\times \frac{\sigma_{2p}^{2}(w-x_{k})\int_{t_{k}}^{t_{k}+l_{k}}\mu_{2}(\rho -\tau ;\vartheta_{2})d\rho +\lambda_{2p}\sigma_{2}^{2}l_{k}}{\sigma_{2p}^{2}{\left(\int_{t_{k}}^{t_{k}+l_{k}}\mu_{2}(\rho -\tau ;\vartheta_{2})d\rho \right)}^{2}+\sigma_{2}^{2}l_{k}}\right]\times \exp\left[-\frac{{\left(w-x_{k}-\lambda_{2p}\int_{t_{k}}^{t_{k}+l_{k}}\mu_{2}(\rho -\tau ;\vartheta_{2})d\rho \right)}^{2}}{2\left({\left(\int_{t_{k}}^{t_{k}+l_{k}}\mu_{2}(\rho -\tau ;\vartheta_{2})d\rho \right)}^{2}\sigma_{2p}^{2}+\sigma_{2}^{2}l_{k}\right)}\right] \end{array}$$

The result of Equation (A3) is transformed from lifetime T to RUL based on the law of total probability as $f_{T}(t)=\int_{-\infty }^{+\infty }f_{T}(t|\lambda_{2},x_{\tau })p(\lambda_{2})d\lambda_{2}$, where the result of $f_{T}(t|\lambda_{2},x_{\tau })$ can be found in Equation (6), and the proof can be found in reference [33]. In this way, the proof has been completed. □

## Appendix B. Proof of Theorem 2

**Proof.**  Case 1: (1) If $t_{k}\leq \tau$ and $0<l_{k}+t_{k}\leq \tau$, To facilitate calculation, the following Lemma A1 is provided. **Lemma A1 ** **([19]).** *If* $Z\sim N(\mu ,\sigma^{2})$ *and* $w_{1},w_{2},A,B\in R^{+}$*, then*

(A4) $$E_{Z}\left\{(w_{1}-AZ)\exp\left[-\frac{{\left(w_{2}-BZ\right)}^{2}}{2C}\right]\right\}=\sqrt{\frac{C}{B^{2}\sigma^{2}+C}}\left(w_{1}-A\frac{Bw_{2}\sigma^{2}+\mu C}{B^{2}\sigma^{2}+C}\right)\exp\left[-\frac{{\left(w_{2}-B\mu \right)}^{2}}{2(B^{2}\sigma^{2}+C)}\right]$$

(A) Based on $f_{T}(t|\lambda_{1})$ in Equation (5) and $\lambda_{1}\sim N(\lambda_{1p},\sigma_{1p}^{2})$, the PDF of the lifetime considering the unit-to-unit variability could be formulated via Lemma A1 as,

(A5) $$\begin{array}{l} f_{T}(t)=\int_{-\infty }^{+\infty }f_{T}(t|\lambda_{1})p(\lambda_{1})d\lambda_{1}\\ ≅\int_{-\infty }^{+\infty }\frac{w-x_{0}-\lambda_{1}\left(\int_{0}^{t}\mu_{1}(\rho ;\vartheta_{1})d\rho -t\mu_{1}(t;\vartheta_{1})\right)}{\sqrt{2\pi \sigma_{1}^{2}t^{3}}}\exp\left[-\frac{{\left(w-x_{0}-\lambda_{1}\int_{0}^{t}\mu_{1}(\rho ;\vartheta_{1})d\rho \right)}^{2}}{2\sigma_{1}^{2}t}\right]p(\lambda_{1})d\lambda_{1}\\ ≅\frac{\left[w-\left(\int_{0}^{t}\mu_{1}(\rho ;\vartheta_{1})d\rho -t\mu_{1}(t;\vartheta_{1})\right)\times \frac{w\sigma_{1p}^{2}\int_{0}^{t}\mu_{1}(\rho ;\vartheta_{1})d\rho +\lambda_{1p}\sigma_{1}^{2}t}{{\left(\int_{0}^{t}\mu_{1}(\rho ;\vartheta_{1})d\rho \right)}^{2}\sigma_{1p}^{2}+\sigma_{1}^{2}t}\right]}{\sqrt{2\pi t^{2}\left({\left(\int_{0}^{t}\mu_{1}(\rho ;\vartheta_{1})d\rho \right)}^{2}\sigma_{1p}^{2}+\sigma_{1}^{2}t\right)}}\times \exp\left[-\frac{{\left(w-\lambda_{1p}\int_{0}^{t}\mu_{1}(\rho ;\vartheta_{1})d\rho \right)}^{2}}{2\left({\left(\int_{0}^{t}\mu_{1}(\rho ;\vartheta_{1})d\rho \right)}^{2}\sigma_{1p}^{2}+\sigma_{1}^{2}t\right)}\right] \end{array}$$

(B) Then based on the relationship between lifetime and RUL, we can further obtain the PDF of RUL considering the unit-to-unit variability as follows,

(A6) $$\begin{array}{l} f_{L}(l_{k})≅\frac{\left[w-x_{k}-\left(\int_{t_{k}}^{t_{k}+l_{k}}\mu_{1}(\rho ;\vartheta_{1})d\rho -l_{k}\mu_{1}(t_{k}+l_{k};\vartheta_{1})\right)\right.\left.\times \frac{(w-x_{k})\sigma_{1p}^{2}\int_{t_{k}}^{t_{k}+l_{k}}\mu_{1}(\rho ;\vartheta_{1})d\rho +\lambda_{1p}\sigma_{1}^{2}l_{k}}{{\left(\int_{t_{k}}^{t_{k}+l_{k}}\mu_{1}(\rho ;\vartheta_{1})d\rho \right)}^{2}\sigma_{1p}^{2}+\sigma_{1}^{2}l_{k}}\right]}{\sqrt{2\pi l_{k}{}^{2}\left({\left(\int_{t_{k}}^{t_{k}+l_{k}}\mu_{1}(\rho ;\vartheta_{1})d\rho \right)}^{2}\sigma_{1p}^{2}+\sigma_{1}^{2}l_{k}\right)}}\\ \times \exp\left[-\frac{{\left(w-x_{k}-\lambda_{1p}\int_{t_{k}}^{t_{k}+l_{k}}\mu_{1}(\rho ;\vartheta_{1})d\rho \right)}^{2}}{2\left({\left(\int_{t_{k}}^{t_{k}+l_{k}}\mu_{1}(\rho ;\vartheta_{1})d\rho \right)}^{2}\sigma_{1p}^{2}+\sigma_{1}^{2}l_{k}\right)}\right] \end{array}$$

(C) When considering the three-source variability simultaneously, it is necessary to further incorporate the measurement variability into the PDF of RUL that considers unit-to-unit variability, which could be presented as,

(A7) $$\begin{array}{l} f_{L_{k}|Y_{1:k}}(l_{k}|Y_{1:k})≅E_{x_{k}|Y_{1:k}}\left\{\frac{\left[w-x_{k}-\left(\int_{t_{k}}^{t_{k}+l_{k}}\mu_{1}(\rho ;\vartheta_{1})d\rho -l_{k}\mu_{1}(t_{k}+l_{k};\vartheta_{1})\right)\right.\left.\times \frac{(w-x_{k})\sigma_{1p}^{2}\int_{t_{k}}^{t_{k}+l_{k}}\mu_{1}(\rho ;\vartheta_{1})d\rho +\lambda_{1p}\sigma_{1}^{2}l_{k}}{{\left(\int_{t_{k}}^{t_{k}+l_{k}}\mu_{1}(\rho ;\vartheta_{1})d\rho \right)}^{2}\sigma_{1p}^{2}+\sigma_{1}^{2}l_{k}}\right]}{\sqrt{2\pi l_{k}{}^{2}\left({\left(\int_{t_{k}}^{t_{k}+l_{k}}\mu_{1}(\rho ;\vartheta_{1})d\rho \right)}^{2}\sigma_{1p}^{2}+\sigma_{1}^{2}l_{k}\right)}}\right.\\ \left.\times \exp\left[-\frac{{\left(w-x_{k}-\lambda_{1p}\int_{t_{k}}^{t_{k}+l_{k}}\mu_{1}(\rho ;\vartheta_{1})d\rho \right)}^{2}}{2\left({\left(\int_{t_{k}}^{t_{k}+l_{k}}\mu_{1}(\rho ;\vartheta_{1})d\rho \right)}^{2}\sigma_{1p}^{2}+\sigma_{1}^{2}l_{k}\right)}\right]\right\} \end{array}$$

(D) Then, based on $x_{k}\sim N({\hat{x}}_{k|k},P_{k|k})$ and Lemma A1, we can obtain the PDF of RUL with three-source variability when $t_{k}\leq \tau$ and $0<l_{k}+t_{k}\leq \tau$ as follows,

(A8) $$f_{L_{k}|Y_{1:k}}(l_{k}|Y_{1:k})≅\frac{M_{1}-N_{1}\times \frac{\left(w-\lambda_{1p}\int_{t_{k}}^{t_{k}+l_{k}}\mu_{1}(\rho ;\vartheta_{1})d\rho \right)P_{k|k}+\upsilon_{1}(l_{k}){\hat{x}}_{k|k}}{P_{k|k}+\upsilon_{1}(l_{k})}}{\sqrt{2\pi l_{k}{}^{2}\upsilon_{1}^{2}(l_{k})\left(P_{k|k}+\upsilon_{1}(l_{k})\right)}}\exp\left[-\frac{{\left(w-{\hat{x}}_{k|k}-\lambda_{1p}\int_{t_{k}}^{t_{k}+l_{k}}\mu_{1}(\rho ;\vartheta_{1})d\rho \right)}^{2}}{2\left(P_{k|k}+\upsilon_{1}(l_{k})\right)}\right]$$

where,

$$\begin{array}{l} \upsilon_{1}(l_{k})={\left(\int_{t_{k}}^{t_{k}+l_{k}}\mu_{1}(\rho ;\vartheta_{1})d\rho \right)}^{2}\sigma_{1p}^{2}+\sigma_{1}^{2}l_{k},\\ M_{1}=w\upsilon_{1}(l_{k})-\left(w\sigma_{1p}^{2}\int_{t_{k}}^{t_{k}+l_{k}}\mu_{1}(\rho ;\vartheta_{1})d\rho +\lambda_{1p}\sigma_{1}^{2}l_{k}\right)\left(\int_{t_{k}}^{t_{k}+l_{k}}\mu_{1}(\rho ;\vartheta_{1})d\rho -l_{k}\mu_{1}(t_{k}+l_{k};\vartheta_{1})\right),\\ N_{1}=\upsilon_{1}(l_{k})-\sigma_{1p}^{2}\int_{t_{k}}^{t_{k}+l_{k}}\mu_{1}(\rho ;\vartheta_{1})d\rho \left(\int_{t_{k}}^{t_{k}+l_{k}}\mu_{1}(\rho ;\vartheta_{1})d\rho -l_{k}\mu_{1}(t_{k}+l_{k};\vartheta_{1})\right) \end{array}$$

(2) If $t_{k}\leq \tau$ and $\tau <l_{k}+t_{k}$, (A) To solve the PDF of RUL considering three-source variability simultaneously, the PDF formulas in S−T of Theorem 1 can be transformed into the following form,

(A9) $$\begin{array}{l} S=S_{1}-S_{2}\\ =\frac{1}{\sqrt{2\pi {\left(t_{k}+l_{k}-\tau \right)}^{2}(\sigma_{\alpha 2}^{2}+\sigma_{\beta 2}^{2})}}\exp\left[-\frac{{\left(\lambda_{\alpha 2}-\lambda_{\beta 2}\right)}^{2}}{2(\sigma_{\alpha 2}^{2}+\sigma_{\beta 2}^{2})}\right]\\ \times \left\{\left(r_{a2}\times \frac{\lambda_{\beta 2}\sigma_{\alpha 2}^{2}+\lambda_{\alpha 2}\sigma_{\beta 2}^{2}}{\sigma_{\alpha 2}^{2}+\sigma_{\beta 2}^{2}}-r_{b2}\right)\times \Phi \left(\frac{\lambda_{\beta 2}\sigma_{\alpha 2}^{2}+\lambda_{\alpha 2}\sigma_{\beta 2}^{2}}{\sqrt{\sigma_{\alpha 2}^{2}\sigma_{\beta 2}^{2}(\sigma_{\alpha 2}^{2}+\sigma_{\beta 2}^{2})}}\right)+r_{a2}\sqrt{\frac{\sigma_{\alpha 2}^{2}\sigma_{\beta 2}^{2}}{\sigma_{\alpha 2}^{2}+\sigma_{\beta 2}^{2}}}\times \phi \left(\frac{\lambda_{\beta 2}\sigma_{\alpha 2}^{2}+\lambda_{\alpha 2}\sigma_{\beta 2}^{2}}{\sqrt{\sigma_{\alpha 2}^{2}\sigma_{\beta 2}^{2}(\sigma_{\alpha 2}^{2}+\sigma_{\beta 2}^{2})}}\right)\right\}\\ T=T_{1}-T_{2}\\ =\frac{I_{2}}{\sqrt{2\pi {\left(t_{k}+l_{k}-\tau \right)}^{2}(\sigma_{\alpha 2}^{2}+\sigma_{\beta 2}^{2})}}\exp\left[-\frac{{\left(\lambda_{\alpha 2}-\lambda_{\gamma 2}\right)}^{2}}{2(\sigma_{\alpha 2}^{2}+\sigma_{\beta 2}^{2})}\right]\\ \times \left\{\left(r_{a2}\times \frac{\lambda_{\gamma 2}\sigma_{\alpha 2}^{2}+\lambda_{\alpha 2}\sigma_{\beta 2}^{2}}{\sigma_{\alpha 2}^{2}+\sigma_{\beta 2}^{2}}-r_{b2}\right)\times \Phi \left(\frac{\lambda_{\gamma 2}\sigma_{\alpha 2}^{2}+\lambda_{\alpha 2}\sigma_{\beta 2}^{2}}{\sqrt{\sigma_{\alpha 2}^{2}\sigma_{\beta 2}^{2}(\sigma_{\alpha 2}^{2}+\sigma_{\beta 2}^{2})}}\right)+r_{a2}\sqrt{\frac{\sigma_{\alpha 2}^{2}\sigma_{\beta 2}^{2}}{\sigma_{\alpha 2}^{2}+\sigma_{\beta 2}^{2}}}\times \phi \left(\frac{\lambda_{\gamma 2}\sigma_{\alpha 2}^{2}+\lambda_{\alpha 2}\sigma_{\beta 2}^{2}}{\sqrt{\sigma_{\alpha 2}^{2}\sigma_{\beta 2}^{2}(\sigma_{\alpha 2}^{2}+\sigma_{\beta 2}^{2})}}\right)\right\} \end{array}$$

where

$$\begin{array}{l} \lambda_{\alpha 2}=\lambda_{2p}\int_{\tau }^{t_{k}+l_{k}}\mu_{2}(\rho -\tau ;\vartheta_{2})d\rho ,\lambda_{\beta 2}=w-x_{k}-\lambda_{1p}\int_{t_{k}}^{\tau }\mu_{1}(\rho ;\vartheta_{1})d\rho ,\lambda_{\gamma 2}=-w+x_{k}-\lambda_{1p}\int_{t_{k}}^{\tau }\mu_{1}(\rho ;\vartheta_{1})d\rho -\frac{2(w-x_{k})\sigma_{1p}^{2}\int_{t_{k}}^{\tau }\mu_{1}(\rho ;\vartheta_{1})d\rho }{\sigma_{1}^{2}},\\ \sigma_{\alpha 2}^{2}=\sigma_{2p}^{2}{\left(\int_{\tau }^{t_{k}+l_{k}}\mu_{2}(\rho -\tau ;\vartheta_{2})d\rho \right)}^{2}+\sigma_{2}^{2}(t_{k}+l_{k}-\tau ),\sigma_{\beta 2}^{2}=\sigma_{1p}^{2}{\left(\int_{t_{k}}^{\tau }\mu_{1}(\rho ;\vartheta_{1})d\rho \right)}^{2}+\sigma_{1}^{2}(\tau -t_{k}),\\ r_{a2}=\frac{(t_{k}+l_{k}-\tau )\left(\sigma_{2}^{2}+\sigma_{2p}^{2}\mu_{2}(t_{k}+l_{k}-\tau ;\vartheta_{2})\int_{\tau }^{t_{k}+l_{k}}\mu_{2}(\rho -\tau ;\vartheta_{2})d\rho \right)}{\sigma_{\alpha 2}^{2}},r_{b2}=\frac{(t_{k}+l_{k}-\tau )\sigma_{2}^{2}\left(\lambda_{a2}-\lambda_{2p}(t_{k}+l_{k}-\tau )\mu_{2}(t_{k}+l_{k}-\tau ;\vartheta_{2})\right)}{\sigma_{\alpha 2}^{2}},\\ I_{2}=\exp\left[\frac{2\lambda_{1p}(w-x_{k})}{\sigma_{1}^{2}}+\frac{2\left({\left(w-x_{k}\right)}^{2}\sigma_{1p}^{4}\int_{t_{k}}^{\tau }\mu_{1}(\rho ;\vartheta_{1})d\rho +{\left(w-x_{k}\right)}^{2}\sigma_{1p}^{2}\sigma_{1}^{2}\right)}{\left(\sigma_{1}^{2}+\sigma_{1p}^{2}\int_{t_{k}}^{\tau }\mu_{1}(\rho ;\vartheta_{1})d\rho \right)\sigma_{1}^{4}}\right] \end{array}$$

(B) Then, based on Equation (A9) and $x_{k}\sim N({\hat{x}}_{k|k},P_{k|k})$, the PDF of RUL with three-source variability can be formulated as follows,

(A10) $$f_{L_{k}|Y_{1:k}}(l_{k}|Y_{1:k})=E_{x_{k}|Y_{1:k}}\left[S-T\right]=E_{x_{k}|Y_{1:k}}\left[S\right]-E_{x_{k}|Y_{1:k}}\left[T\right]$$

(C) Equation (A10) can be obtained through solving $E_{x_{k}|Y_{1:k}}\left[S\right]$ and $E_{x_{k}|Y_{1:k}}\left[T\right]$ separately. To facilitate calculation, the following Lemmas A2 and A3 are provided. **Lemma A2** **([45]).** *If* $Z\sim N(\mu ,\sigma^{2})$, w,A∈R *and* $B\in R^{+}$*,* 

(A11) $$E_{Z}\left\{\exp\left[-\frac{{\left(w-AZ\right)}^{2}}{2B}\right]\right\}=\sqrt{\frac{B}{A^{2}\sigma^{2}+B}}\exp\left[-\frac{{\left(w-A\mu \right)}^{2}}{2(A^{2}\sigma^{2}+B)}\right]$$

**Lemma** **A3** **([19]).** *If* $Z\sim N(\mu ,\sigma^{2})$*,* w,A,B,C,D∈R *and* $1-2B\sigma^{2}>0$*, then,*

(A12) $$E_{Z}\left[\exp({\mathrm{AZ}+\mathrm{BZ}}^{2})\cdot \Phi (C+\mathrm{DZ})\right]=\frac{1}{\sqrt{1-2B\sigma^{2}}}\exp\left[\frac{2A\mu +A^{2}\sigma^{2}+2B\mu^{2}}{2\left(1-2B\sigma^{2}\right)}\right]\cdot \Phi \left(\frac{C+D\mu +AD\sigma^{2}-2BC\sigma^{2}}{\sqrt{{\left(1-2B\sigma^{2}\right)}^{2}+D^{2}\sigma^{2}\left(1-2B\sigma^{2}\right)}}\right)$$

(D) Then, to calculate $E_{x_{k}|Y_{1:k}}\left[S\right]$, the S formula in Equation (A9) is expanded to $S_{a}$ and $S_{b}$ as follows,

(A13) $$\begin{array}{l} S_{a}=\exp\left[-\frac{{\left(\lambda_{\alpha 2}-\lambda_{\beta 2}\right)}^{2}}{2(\sigma_{\alpha 2}^{2}+\sigma_{\beta 2}^{2})}\right]\times \left(r_{a2}\times \frac{\lambda_{\beta 2}\sigma_{\alpha 2}^{2}+\lambda_{\alpha 2}\sigma_{\beta 2}^{2}}{\sigma_{\alpha 2}^{2}+\sigma_{\beta 2}^{2}}-r_{b2}\right)\times \Phi \left(\frac{\lambda_{\beta 2}\sigma_{\alpha 2}^{2}+\lambda_{\alpha 2}\sigma_{\beta 2}^{2}}{\sqrt{\sigma_{\alpha 2}^{2}\sigma_{\beta 2}^{2}(\sigma_{\alpha 2}^{2}+\sigma_{\beta 2}^{2})}}\right)\\ =\exp\left[-\frac{{\left(\lambda_{2p}\int_{\tau }^{t_{k}+l_{k}}\mu_{2}(\rho -\tau ;\vartheta_{2})d\rho -\left(w-x_{k}-\lambda_{1p}\int_{t_{k}}^{\tau }\mu_{1}(\rho ;\vartheta_{1})d\rho \right)\right)}^{2}}{2(\sigma_{\alpha 2}^{2}+\sigma_{\beta 2}^{2})}\right]\\ \times \left(r_{a2}\times \frac{\left(w-x_{k}-\lambda_{1p}\int_{t_{k}}^{\tau }\mu_{1}(\rho ;\vartheta_{1})d\rho \right)\sigma_{\alpha 2}^{2}+\left(\lambda_{2p}\int_{\tau }^{t_{k}+l_{k}}\mu_{2}(\rho -\tau ;\vartheta_{2})d\rho \right)\sigma_{\beta 2}^{2}}{\sigma_{\alpha 2}^{2}+\sigma_{\beta 2}^{2}}-r_{b2}\right)\\ \times \Phi \left(\frac{\left(w-x_{k}-\lambda_{1p}\int_{t_{k}}^{\tau }\mu_{1}(\rho ;\vartheta_{1})d\rho \right)\sigma_{\alpha 2}^{2}+\left(\lambda_{2p}\int_{\tau }^{t_{k}+l_{k}}\mu_{2}(\rho -\tau ;\vartheta_{2})d\rho \right)\sigma_{\beta 2}^{2}}{\sqrt{\sigma_{\alpha 2}^{2}\sigma_{\beta 2}^{2}(\sigma_{\alpha 2}^{2}+\sigma_{\beta 2}^{2})}}\right) \end{array}$$

$$\begin{array}{l} S_{b}=\exp\left[-\frac{{\left(\lambda_{\alpha 2}-\lambda_{\beta 2}\right)}^{2}}{2(\sigma_{\alpha 2}^{2}+\sigma_{\beta 2}^{2})}\right]\times \phi \left(\frac{\lambda_{\beta 2}\sigma_{\alpha 2}^{2}+\lambda_{\alpha 2}\sigma_{\beta 2}^{2}}{\sqrt{\sigma_{\alpha 2}^{2}\sigma_{\beta 2}^{2}(\sigma_{\alpha 2}^{2}+\sigma_{\beta 2}^{2})}}\right)\\ =\exp\left[-\frac{{\left(\lambda_{2p}\int_{\tau }^{t_{k}+l_{k}}\mu_{2}(\rho -\tau ;\vartheta_{2})d\rho -\left(w-x_{k}-\lambda_{1p}\int_{t_{k}}^{\tau }\mu_{1}(\rho ;\vartheta_{1})d\rho \right)\right)}^{2}}{2(\sigma_{\alpha 2}^{2}+\sigma_{\beta 2}^{2})}\right]\\ \times \phi \left(\frac{\left(w-x_{k}-\lambda_{1p}\int_{t_{k}}^{\tau }\mu_{1}(\rho ;\vartheta_{1})d\rho \right)\sigma_{\alpha 2}^{2}+\left(\lambda_{2p}\int_{\tau }^{t_{k}+l_{k}}\mu_{2}(\rho -\tau ;\vartheta_{2})d\rho \right)\sigma_{\beta 2}^{2}}{\sqrt{\sigma_{\alpha 2}^{2}\sigma_{\beta 2}^{2}(\sigma_{\alpha 2}^{2}+\sigma_{\beta 2}^{2})}}\right) \end{array}$$

where

$$\begin{array}{l} \sigma_{\alpha 2}^{2}=\sigma_{2p}^{2}{\left(\int_{\tau }^{t_{k}+l_{k}}\mu_{2}(\rho -\tau ;\vartheta_{2})d\rho \right)}^{2}+\sigma_{2}^{2}(t_{k}+l_{k}-\tau ),\sigma_{\beta 2}^{2}=\sigma_{1p}^{2}{\left(\int_{t_{k}}^{\tau }\mu_{1}(\rho ;\vartheta_{1})d\rho \right)}^{2}+\sigma_{1}^{2}(\tau -t_{k}),\\ r_{a2}=\frac{(t_{k}+l_{k}-\tau )\left(\sigma_{2}^{2}+\sigma_{2p}^{2}\mu_{2}(t_{k}+l_{k}-\tau ;\vartheta_{2})\int_{\tau }^{t_{k}+l_{k}}\mu_{2}(\rho -\tau ;\vartheta_{2})d\rho \right)}{\sigma_{\alpha 2}^{2}},\\ r_{b2}=\frac{(t_{k}+l_{k}-\tau )\sigma_{2}^{2}\left(\lambda_{2p}\int_{\tau }^{t_{k}+l_{k}}\mu_{2}(\rho -\tau ;\vartheta_{2})d\rho -\lambda_{2p}(t_{k}+l_{k}-\tau )\mu_{2}(t_{k}+l_{k}-\tau ;\vartheta_{2})\right)}{\sigma_{\alpha 2}^{2}}, \end{array}$$

(E) Based on Equation (A13), $E_{x_{k}|Y_{1:k}}\left[S\right]$ could be expressed as follows,

(A14) $$E_{x_{k}|Y_{1:k}}\left[S\right]=\frac{1}{\sqrt{2\pi {\left(t_{k}+l_{k}-\tau \right)}^{2}(\sigma_{\alpha 2}^{2}+\sigma_{\beta 2}^{2})}}\times \left\{E_{x_{k}|Y_{1:k}}\left[S_{a}\right]+r_{a2}\sqrt{\frac{\sigma_{\alpha 2}^{2}\sigma_{\beta 2}^{2}}{\sigma_{\alpha 2}^{2}+\sigma_{\beta 2}^{2}}}E_{x_{k}|Y_{1:k}}\left[S_{b}\right]\right\}$$

(F) To solve $E_{x_{k}|Y_{1:k}}\left[S_{a}\right]$, we define the following parameter simplification formulas,

(A15) $$\begin{array}{l} a_{S}=w-\lambda_{1p}\int_{t_{k}}^{\tau }\mu_{1}(\rho ;\vartheta_{1})d\rho -\lambda_{2p}\int_{\tau }^{t_{k}+l_{k}}\mu_{2}(\rho -\tau ;\vartheta_{2})d\rho ,b_{S}=\sigma_{\alpha 2}^{2}+\sigma_{\beta 2}^{2},c_{S}=-r_{a2}\times \frac{\sigma_{\alpha 2}^{2}}{\sigma_{\alpha 2}^{2}+\sigma_{\beta 2}^{2}},\\ d_{S}=r_{a2}\times \frac{\left(w-\lambda_{1p}\int_{t_{k}}^{\tau }\mu_{1}(\rho ;\vartheta_{1})d\rho \right)\sigma_{\alpha 2}^{2}+\left(\lambda_{2p}\int_{\tau }^{t_{k}+l_{k}}\mu_{2}(\rho -\tau ;\vartheta_{2})d\rho \right)\sigma_{\beta 2}^{2}}{\sigma_{\alpha 2}^{2}+\sigma_{\beta 2}^{2}}-r_{b2},\\ e_{S}=\left(w-\lambda_{1p}\int_{t_{k}}^{\tau }\mu_{1}(\rho ;\vartheta_{1})d\rho \right)\sigma_{\alpha 2}^{2}+\left(\lambda_{2p}\int_{\tau }^{t_{k}+l_{k}}\mu_{2}(\rho -\tau ;\vartheta_{2})d\rho \right)\sigma_{\beta 2}^{2},f_{S}=\sqrt{\sigma_{\alpha 2}^{2}\sigma_{\beta 2}^{2}(\sigma_{\alpha 2}^{2}+\sigma_{\beta 2}^{2})} \end{array}$$

Then, the formula $S_{a}$ in Equation (A13) could be simplified as,

(A16) $$S_{a}=\exp\left[-\frac{{\left(a_{S}-x_{k}\right)}^{2}}{2b_{S}}\right]\times \left(d_{S}+c_{S}x_{k}\right)\times \Phi \left(\frac{e_{S}-\sigma_{\alpha 2}^{2}x_{k}}{f_{S}}\right)$$

(G) Thus, based on $x_{k}\sim N({\hat{x}}_{k|k},P_{k|k})$ and Lemma A3, $E_{x_{k}|Y_{1:k}}\left[S_{a}\right]$ could be obtained as follows,

(A17) $$\begin{array}{l} E_{x_{k}|Y_{1:k}}\left[S_{a}\right]=E_{x_{k}|Y_{1:k}}\left[\exp\left[-\frac{{\left(a_{S}-x_{k}\right)}^{2}}{2b_{S}}\right]\times d_{S}\times \Phi \left(\frac{e_{S}-\sigma_{\alpha 2}^{2}x_{k}}{f_{S}}\right)\right]\\ +E_{x_{k}|Y_{1:k}}\left[\exp\left[-\frac{{\left(a_{S}-x_{k}\right)}^{2}}{2b_{S}}\right]\times \left(c_{S}x_{k}\right)\times \Phi \left(\frac{e_{S}-\sigma_{\alpha 2}^{2}x_{k}}{f_{S}}\right)\right]\\ =\frac{d_{S}\exp\left[-\frac{a_{S}^{2}}{2b_{S}}\right]}{\sqrt{1+\frac{P_{k|k}}{b_{S}}}}\exp\left[\frac{2a_{S}{\hat{x}}_{k|k}+\frac{a_{S}^{2}P_{k|k}}{b_{3}}-{\hat{x}}_{k|k}^{2}}{2\left(b_{S}+P_{k|k}\right)}\right]\Phi \left(\frac{e_{S}-\sigma_{\alpha 2}^{2}{\hat{x}}_{k|k}-\frac{a_{S}\sigma_{\alpha 2}^{2}P_{k|k}}{b_{S}}+\frac{e_{S}P_{k|k}}{b_{S}}}{\sqrt{f_{S}^{2}{\left(1+\frac{P_{k|k}}{b_{S}}\right)}^{2}+\sigma_{\alpha 2}^{4}P_{k|k}\left(1+\frac{P_{k|k}}{b_{S}}\right)}}\right)\\ +\frac{1}{\sqrt{2\pi P_{k|k}}}\int_{-\infty }^{+\infty }\left(\exp\left[-\frac{{\left(a_{S}-x_{k}\right)}^{2}}{2b_{S}}\right]\times \left(c_{S}x_{k}\right)\times \exp\left[-\frac{{\left(x_{k}-{\hat{x}}_{k|k}\right)}^{2}}{2P_{k|k}}\right]\times \Phi \left(\frac{e_{S}-\sigma_{\alpha 2}^{2}x_{k}}{f_{S}}\right)\right)dx_{k} \end{array}$$

(H) To solve $E_{x_{k}|Y_{1:k}}\left[S_{b}\right]$, based on Equation (15), the $S_{b}$ in Equation (A13) could be simplified as,

(A18) $$S_{b}=\exp\left[-\frac{{\left(a_{S}-x_{k}\right)}^{2}}{2b_{S}}\right]\times \frac{1}{\sqrt{2\pi }}\exp\left[-\frac{{\left(e_{S}-\sigma_{\alpha 2}^{2}x_{k}\right)}^{2}}{2f_{S}^{2}}\right]$$

Thus, $E_{x_{k}|Y_{1:k}}\left[S_{b}\right]$ could be formulated as follows,

(A19) $$\begin{array}{l} E_{x_{k}|Y_{1:k}}\left[S_{b}\right]=E_{x_{k}|Y_{1:k}}\left\{\exp\left[-\frac{{\left(a_{S}-x_{k}\right)}^{2}}{2b_{S}}\right]\times \frac{1}{\sqrt{2\pi }}\exp\left[-\frac{{\left(e_{S}-\sigma_{\alpha 2}^{2}x_{k}\right)}^{2}}{2f_{S}^{2}}\right]\right\}\\ =\frac{1}{\sqrt{2\pi }}E_{x_{k}|Y_{1:k}}\left\{\exp\left[-\frac{1}{2}\left(\frac{f_{S}^{2}+b_{S}\sigma_{\alpha 2}^{4}}{b_{S}f_{S}^{2}}x_{k}^{2}-\frac{2\left(a_{S}f_{S}^{2}+b_{S}e_{S}\sigma_{\alpha 2}^{2}\right)}{b_{S}f_{S}^{2}}x_{k}+\frac{a_{S}^{2}f_{S}^{2}+b_{S}e_{S}^{2}}{b_{S}f_{S}^{2}}\right)\right]\right\}\\ =\frac{1}{\sqrt{2\pi }}E_{x_{k}|Y_{1:k}}\left\{\exp\left[-\frac{1}{2}\left({\left(L_{1S}x_{k}-L_{2S}\right)}^{2}+L_{3S}\right)\right]\right\} \end{array}$$

where

$$L_{1S}=\sqrt{\frac{f_{S}^{2}+b_{S}\sigma_{\alpha 2}^{4}}{b_{S}f_{S}^{2}}},L_{2S}=\frac{a_{S}f_{S}^{2}+b_{S}e_{S}\sigma_{\alpha 2}^{4}}{\sqrt{b_{S}f_{S}^{2}\left(f_{S}^{2}+b_{S}\sigma_{\alpha 2}^{4}\right)}},L_{3S}=\frac{a_{S}^{2}f_{S}^{2}+b_{S}e_{S}^{2}}{b_{S}f_{S}^{2}}-\frac{{\left(a_{S}f_{S}^{2}+b_{S}e_{S}\sigma_{\alpha 2}^{2}\right)}^{2}}{\sqrt{b_{S}f_{S}^{2}\left(f_{S}^{2}+b_{S}\sigma_{\alpha 2}^{4}\right)}}$$

Then, according to Lemma A2, $E_{x_{k}|Y_{1:k}}\left[S_{b}\right]$ could be obtained as,

(A20) $$\begin{array}{l} E_{x_{k}|Y_{1:k}}\left[S_{b}\right]=\frac{1}{\sqrt{2\pi }}E_{x_{k}|Y_{1:k}}\left[\exp\left(-\frac{1}{2}\left({\left(L_{1S}x_{k}-L_{2S}\right)}^{2}+L_{3S}\right)\right)\right]\\ =\frac{1}{\sqrt{2\pi }}\exp\left(-\frac{1}{2}L_{3S}\right)\times E_{x_{k}|Y_{1:k}}\left[\exp\left(-\frac{1}{2}{\left(L_{1S}x_{k}-L_{2S}\right)}^{2}\right)\right]\\ =\frac{1}{\sqrt{2\pi }}\exp\left(-\frac{1}{2}L_{3S}\right)\times \sqrt{\frac{1}{L_{1S}^{2}P_{k|k}+1}}\exp\left[-\frac{{\left(L_{2S}-L_{1S}{\hat{x}}_{k|k}\right)}^{2}}{2\left(L_{1S}^{2}P_{k|k}+1\right)}\right] \end{array}$$

(I) Finally, bringing the results of $E_{x_{k}|Y_{1:k}}\left[S_{a}\right]$ and $E_{x_{k}|Y_{1:k}}\left[S_{b}\right]$ into Equation (A14), the final result of $E_{x_{k}|Y_{1:k}}\left[S\right]$, i.e., the formula $S_{new}$ in Theorem 2, could be obtained. Due to space limitations, the result of $S_{new}$ is omitted here. Similarly, we can solve $E_{x_{k}|Y_{1:k}}\left[T\right]$ in Equation (A10) as follows. (A) Firstly, $I_{2}$ in the formula T of Equation (A9) should be simplified as,

(A21) $$\begin{array}{l} I_{2}=\exp\left[\frac{2\lambda_{1p}(w-x_{k})}{\sigma_{1}^{2}}+\frac{2\left({\left(w-x_{k}\right)}^{2}\sigma_{1p}^{4}\int_{t_{k}}^{\tau }\mu_{1}(\rho ;\vartheta_{1})d\rho +{\left(w-x_{k}\right)}^{2}\sigma_{1p}^{2}\sigma_{1}^{2}\right)}{\left(\sigma_{1}^{2}+\sigma_{1p}^{2}\int_{t_{k}}^{\tau }\mu_{1}(\rho ;\vartheta_{1})d\rho \right)\sigma_{1}^{4}}\right]\\ =\exp\left[\frac{2w\left(\lambda_{1p}\sigma_{1}^{2}+\sigma_{1p}^{2}w\right)}{\sigma_{1}^{4}}\right]\exp\left[\frac{2\sigma_{1p}^{2}}{\sigma_{1}^{4}}x_{k}^{2}-\frac{2\lambda_{1p}\sigma_{1}^{2}+4w\sigma_{1p}^{2}}{\sigma_{1}^{4}}x_{k}\right]\\ =\exp\left[\frac{2w\left(\lambda_{1p}\sigma_{1}^{2}+\sigma_{1p}^{2}w\right)}{\sigma_{1}^{4}}\right]\exp\left(a_{T}x_{k}^{2}-b_{T}x_{k}\right) \end{array}$$

where $a_{T}=\frac{2\sigma_{1p}^{2}}{\sigma_{1}^{4}},b_{T}=\frac{2\lambda_{1p}\sigma_{1}^{2}+4w\sigma_{1p}^{2}}{\sigma_{1}^{4}}$. (B) Further, as to

(A22) $$\begin{array}{l} \exp\left[-\frac{{\left(\lambda_{\alpha 2}-\lambda_{\gamma 2}\right)}^{2}}{2(\sigma_{\alpha 2}^{2}+\sigma_{\beta 2}^{2})}\right]\\ =\exp\left[-\frac{{\left[\lambda_{2p}\int_{\tau }^{t_{k}+l_{k}}\mu_{2}(\rho -\tau ;\vartheta_{2})d\rho +w-x_{k}+\lambda_{1p}\int_{t_{k}}^{\tau }\mu_{1}(\rho ;\vartheta_{1})d\rho +\frac{2(w-x_{k})\sigma_{1p}^{2}\left(\int_{t_{k}}^{\tau }\mu_{1}(\rho ;\vartheta_{1})d\rho \right)}{\sigma_{1}^{2}}\right]}^{2}}{2(\sigma_{\alpha 2}^{2}+\sigma_{\beta 2}^{2})}\right] \end{array}$$

we define

(A23) $$\begin{array}{l} c_{T}=w+\lambda_{1p}\int_{t_{k}}^{\tau }\mu_{1}(\rho ;\vartheta_{1})d\rho +\lambda_{2p}\int_{\tau }^{t_{k}+l_{k}}\mu_{2}(\rho -\tau ;\vartheta_{2})d\rho +\frac{2w\sigma_{1p}^{2}\int_{t_{k}}^{\tau }\mu_{1}(\rho ;\vartheta_{1})d\rho }{\sigma_{1}^{2}}\\ d_{T}=1+\frac{2\sigma_{1p}^{2}\int_{t_{k}}^{\tau }\mu_{1}(\rho ;\vartheta_{1})d\rho }{\sigma_{1}^{2}}=\frac{\sigma_{1}^{2}+2\sigma_{1p}^{2}\int_{t_{k}}^{\tau }\mu_{1}(\rho ;\vartheta_{1})d\rho }{\sigma_{1}^{2}} \end{array}$$

Thus, the Equation (A22) could be simplified as,

(A24) $$\exp\left[-\frac{{\left(c_{T}-d_{T}x_{k}\right)}^{2}}{2b_{S}}\right]$$

Based on Equations (A21) and (A24), we have

(A25) $$\begin{array}{l} I_{2}\exp\left[-\frac{{\left(\lambda_{\alpha 2}-\lambda_{\gamma 2}\right)}^{2}}{2(\sigma_{\alpha 2}^{2}+\sigma_{\beta 2}^{2})}\right]\\ =\exp\left[\frac{2w\left(\lambda_{1p}\sigma_{1}^{2}+\sigma_{1p}^{2}w\right)}{\sigma_{1}^{4}}\right]\exp\left(a_{T}x_{k}^{2}-b_{T}x_{k}\right)\times \exp\left[-\frac{{\left(c_{T}-d_{T}x_{k}\right)}^{2}}{2b_{S}}\right]\\ =\exp\left[\frac{2w\left(\lambda_{1p}\sigma_{1}^{2}+\sigma_{1p}^{2}w\right)}{\sigma_{1}^{4}}\right]\exp\left[-\frac{d_{T}^{2}-2a_{T}b_{S}}{2b_{S}}x_{k}^{2}+\frac{c_{T}d_{T}-b_{S}b_{T}}{b_{S}}x_{k}-\frac{c_{T}^{2}}{2b_{S}}\right]\\ =\exp\left[\frac{2w\left(\lambda_{1p}\sigma_{1}^{2}+\sigma_{1p}^{2}w\right)}{\sigma_{1}^{4}}\right]\exp\left[-\frac{c_{T}^{2}}{2b_{S}}\right]\exp\left[-\frac{D_{1T}}{2b_{S}}x_{k}^{2}+\frac{D_{2T}}{b_{S}}x_{k}\right] \end{array}$$

where $D_{1T}=d_{T}^{2}-2a_{T}b_{S},D_{2T}=c_{T}d_{T}-b_{S}b_{T}$. (C) Then, the common item before $\left\{\cdot \right\}$ in the formula T of Equation (A9) could be simplified as follows,

(A26) $$\begin{array}{l} \frac{I_{2}}{\sqrt{2\pi {\left(t_{k}+l_{k}-\tau \right)}^{2}(\sigma_{\alpha 2}^{2}+\sigma_{\beta 2}^{2})}}\exp\left[-\frac{{\left(\lambda_{\alpha 2}-\lambda_{\gamma 2}\right)}^{2}}{2(\sigma_{\alpha 2}^{2}+\sigma_{\beta 2}^{2})}\right]\\ =\frac{\exp\left[\frac{2w\left(\lambda_{1p}\sigma_{1}^{2}+\sigma_{1p}^{2}w\right)}{\sigma_{1}^{4}}\right]\exp\left[-\frac{c_{T}^{2}}{2b_{S}}\right]\exp\left[-\frac{D_{1T}}{2b_{S}}x_{k}^{2}+\frac{D_{2T}}{b_{S}}x_{k}\right]}{\sqrt{2\pi {\left(t_{k}+l_{k}-\tau \right)}^{2}(\sigma_{\alpha 2}^{2}+\sigma_{\beta 2}^{2})}}\\ =\frac{\exp\left[\frac{4wb_{S}\left(\lambda_{1p}\sigma_{1}^{2}+\sigma_{1p}^{2}w\right)-c_{T}^{2}\sigma_{1}^{4}}{2b_{S}\sigma_{1}^{4}}\right]}{\sqrt{2\pi {\left(t_{k}+l_{k}-\tau \right)}^{2}(\sigma_{\alpha 2}^{2}+\sigma_{\beta 2}^{2})}}\times \exp\left[-\frac{D_{1T}}{2b_{S}}x_{k}^{2}+\frac{D_{2T}}{b_{S}}x_{k}\right] \end{array}$$

(D) On this basis, the formula T in Equation (A9) could be split into $T_{a}$ and $T_{b}$ as follows,

(A27) $$\begin{array}{l} T_{a}=\exp\left[-\frac{D_{1T}}{2b_{S}}x_{k}^{2}+\frac{D_{2T}}{b_{S}}x_{k}\right]\times \left(r_{a2}\times \frac{\lambda_{\gamma 2}\sigma_{\alpha 2}^{2}+\lambda_{\alpha 2}\sigma_{\beta 2}^{2}}{\sigma_{\alpha 2}^{2}+\sigma_{\beta 2}^{2}}-r_{b2}\right)\times \Phi \left(\frac{\lambda_{\gamma 2}\sigma_{\alpha 2}^{2}+\lambda_{\alpha 2}\sigma_{\beta 2}^{2}}{\sqrt{\sigma_{\alpha 2}^{2}\sigma_{\beta 2}^{2}(\sigma_{\alpha 2}^{2}+\sigma_{\beta 2}^{2})}}\right)\\ =\exp\left[-\frac{D_{1T}}{2b_{S}}x_{k}^{2}+\frac{D_{2T}}{b_{S}}x_{k}\right]\\ \times \left(r_{a2}\times \frac{\left(-w+x_{k}-\lambda_{1p}\int_{t_{k}}^{\tau }\mu_{1}(\rho ;\vartheta_{1})d\rho -\frac{2(w-x_{k})\sigma_{1p}^{2}\int_{t_{k}}^{\tau }\mu_{1}(\rho ;\vartheta_{1})d\rho }{\sigma_{1}^{2}}\right)\sigma_{\alpha 2}^{2}+\lambda_{2p}\int_{\tau }^{t_{k}+l_{k}}\mu_{2}(\rho -\tau ;\vartheta_{2})d\rho \sigma_{\beta 2}^{2}}{\sigma_{\alpha 2}^{2}+\sigma_{\beta 2}^{2}}-r_{b2}\right)\\ \times \Phi \left(\frac{\left(-w+x_{k}-\lambda_{1p}\int_{t_{k}}^{\tau }\mu_{1}(\rho ;\vartheta_{1})d\rho -\frac{2(w-x_{k})\sigma_{1p}^{2}\int_{t_{k}}^{\tau }\mu_{1}(\rho ;\vartheta_{1})d\rho }{\sigma_{1}^{2}}\right)\sigma_{\alpha 2}^{2}+\lambda_{2p}\int_{\tau }^{t_{k}+l_{k}}\mu_{2}(\rho -\tau ;\vartheta_{2})d\rho \sigma_{\beta 2}^{2}}{\sqrt{\sigma_{\alpha 2}^{2}\sigma_{\beta 2}^{2}(\sigma_{\alpha 2}^{2}+\sigma_{\beta 2}^{2})}}\right) \end{array}$$

(A28) $$\begin{array}{l} T_{b}=\exp\left[-\frac{D_{1T}}{2b_{S}}x_{k}^{2}+\frac{D_{2T}}{b_{S}}x_{k}\right]\\ \times \phi \left(\frac{\left(-w+x_{k}-\lambda_{1p}\int_{t_{k}}^{\tau }\mu_{1}(\rho ;\vartheta_{1})d\rho -\frac{2(w-x_{k})\sigma_{1p}^{2}\int_{t_{k}}^{\tau }\mu_{1}(\rho ;\vartheta_{1})d\rho }{\sigma_{1}^{2}}\right)\sigma_{\alpha 2}^{2}+\lambda_{2p}\int_{\tau }^{t_{k}+l_{k}}\mu_{2}(\rho -\tau ;\vartheta_{2})d\rho \sigma_{\beta 2}^{2}}{\sqrt{\sigma_{\alpha 2}^{2}\sigma_{\beta 2}^{2}(\sigma_{\alpha 2}^{2}+\sigma_{\beta 2}^{2})}}\right) \end{array}$$

(E) Thus, the simplified form of $E_{x_{k}|Y_{1:k}}\left[T\right]$ could be obtained based on $T_{a},T_{b}$,

(A29) $$E_{x_{k}|Y_{1:k}}\left[T\right]=\frac{\exp\left[\frac{4wb_{S}\left(\lambda_{1p}\sigma_{1}^{2}+\sigma_{1p}^{2}w\right)-c_{T}^{2}\sigma_{1}^{4}}{2b_{S}\sigma_{1}^{4}}\right]}{\sqrt{2\pi {\left(t_{k}+l_{k}-\tau \right)}^{2}(\sigma_{\alpha 2}^{2}+\sigma_{\beta 2}^{2})}}\times \left\{E_{x_{k}|Y_{1:k}}\left[T_{a}\right]+r_{a2}\times \sqrt{\frac{\sigma_{\alpha 2}^{2}\sigma_{\beta 2}^{2}}{\sigma_{\alpha 2}^{2}+\sigma_{\beta 2}^{2}}}E_{x_{k}|Y_{1:k}}\left[T_{b}\right]\right\}$$

(F) To solve $E_{x_{k}|Y_{1:k}}\left[T_{a}\right]$, we define

(A30) $$\begin{array}{l} e_{T}=\frac{r_{a2}\sigma_{\alpha 2}^{2}\left(\sigma_{1}^{2}+2\sigma_{1p}^{2}\int_{t_{k}}^{\tau }\mu_{1}(\rho ;\vartheta_{1})d\rho \right)}{\sigma_{1}^{2}\left(\sigma_{\alpha 2}^{2}+\sigma_{\beta 2}^{2}\right)},\\ f_{T}=r_{a2}\times \frac{\left(-w-\lambda_{1p}\int_{t_{k}}^{\tau }\mu_{1}(\rho ;\vartheta_{1})d\rho -\frac{2w\sigma_{1p}^{2}\int_{t_{k}}^{\tau }\mu_{1}(\rho ;\vartheta_{1})d\rho }{\sigma_{1}^{2}}\right)\sigma_{\alpha 2}^{2}+\lambda_{2p}\int_{\tau }^{t_{k}+l_{k}}\mu_{2}(\rho -\tau ;\vartheta_{2})d\rho \sigma_{\beta 2}^{2}}{\sigma_{\alpha 2}^{2}+\sigma_{\beta 2}^{2}}-r_{b2},\\ g_{T}=\left(-w-\lambda_{1p}\int_{t_{k}}^{\tau }\mu_{1}(\rho ;\vartheta_{1})d\rho -\frac{2w\sigma_{1p}^{2}\int_{t_{k}}^{\tau }\mu_{1}(\rho ;\vartheta_{1})d\rho }{\sigma_{1}^{2}}\right)\sigma_{\alpha 2}^{2}+\lambda_{2p}\int_{\tau }^{t_{k}+l_{k}}\mu_{2}(\rho -\tau ;\vartheta_{2})d\rho \sigma_{\beta 2}^{2},\\ h_{T}=\frac{\sigma_{\alpha 2}^{2}\left(\sigma_{1}^{2}+2\sigma_{1p}^{2}\int_{t_{k}}^{\tau }\mu_{1}(\rho ;\vartheta_{1})d\rho \right)}{\sigma_{1}^{2}} \end{array}$$

Then, the $E_{x_{k}|Y_{1:k}}\left[T_{a}\right]$ in Equation (A29) could be formulated as follows,

(A31) $$\begin{array}{l} E_{x_{k}|Y_{1:k}}\left[T_{a}\right]=E_{x_{k}|Y_{1:k}}\left\{\exp\left[-\frac{D_{1T}}{2b_{S}}x_{k}^{2}+\frac{D_{2T}}{b_{S}}x_{k}\right]\times \left(f_{T}+e_{T}x_{k}\right)\times \Phi \left(\frac{g_{T}+h_{T}x_{k}}{f_{S}}\right)\right\}\\ =E_{x_{k}|Y_{1:k}}\left[\exp\left[-\frac{D_{1T}}{2b_{S}}x_{k}^{2}+\frac{D_{2T}}{b_{S}}x_{k}\right]\times f_{T}\times \Phi \left(\frac{g_{T}+h_{T}x_{k}}{f_{S}}\right)\right]\\ +E_{x_{k}|Y_{1:k}}\left[\exp\left[-\frac{D_{1T}}{2b_{S}}x_{k}^{2}+\frac{D_{2T}}{b_{S}}x_{k}\right]\times \left(e_{T}x_{k}\right)\times \Phi \left(\frac{g_{T}+h_{T}x_{k}}{f_{S}}\right)\right] \end{array}$$

(G) Based on $x_{k}\sim N({\hat{x}}_{k|k},P_{k|k})$ and Lemma A3, $E_{x_{k}|Y_{1:k}}\left[T_{a}\right]$ could be obtained as,

(A32) $$\begin{array}{l} E_{x_{k}|Y_{1:k}}\left[T_{a}\right]=\frac{f_{T}}{\sqrt{1+\frac{D_{1T}P_{k|k}}{b_{S}}}}\exp\left[\frac{2D_{2T}{\hat{x}}_{k|k}+\frac{D_{2T}^{2}P_{k|k}}{b_{3}}-D_{1T}{\hat{x}}_{k|k}^{2}}{2\left(b_{S}+D_{1T}P_{k|k}\right)}\right]\Phi \left(\frac{g_{T}+h_{T}{\hat{x}}_{k|k}+\frac{D_{2T}h_{T}P_{k|k}}{b_{S}}+\frac{D_{1T}g_{T}P_{k|k}}{b_{S}}}{\sqrt{f_{S}^{2}{\left(1+\frac{D_{1T}P_{k|k}}{b_{S}}\right)}^{2}+h_{T}^{2}P_{k|k}\left(1+\frac{D_{1T}P_{k|k}}{b_{S}}\right)}}\right)\\ +\frac{1}{\sqrt{2\pi P_{k|k}}}\int_{-\infty }^{+\infty }\left\{\exp\left[-\frac{D_{1T}}{2b_{S}}x_{k}^{2}+\frac{D_{2T}}{b_{S}}x_{k}\right]\times \left(e_{T}x_{k}\right)\times \exp\left[-\frac{{\left(x_{k}-{\hat{x}}_{k|k}\right)}^{2}}{2P_{k|k}}\right]\times \Phi \left(\frac{g_{T}+h_{T}x_{k}}{f_{S}}\right)\right\}dx_{k} \end{array}$$

(H) Similarly, according to Equation (A30), $E_{x_{k}|Y_{1:k}}\left[T_{b}\right]$ could be formulated as,

(A33) $$\begin{array}{l} E_{x_{k}|Y_{1:k}}\left[T_{b}\right]=E_{x_{k}|Y_{1:k}}\left[\exp\left[-\frac{D_{1T}}{2b_{S}}x_{k}^{2}+\frac{D_{2T}}{b_{S}}x_{k}\right]\times \frac{1}{\sqrt{2\pi }}\exp\left[-\frac{{\left(g_{T}+h_{T}x_{k}\right)}^{2}}{2f_{S}^{2}}\right]\right]\\ =\frac{1}{\sqrt{2\pi }}E_{x_{k}|Y_{1:k}}\left\{\exp\left[-\frac{1}{2}\left(\frac{D_{1T}f_{S}^{2}+b_{S}h_{T}^{2}}{b_{S}f_{T}^{2}}x_{k}^{2}-\frac{2\left(D_{2T}f_{S}^{2}-b_{S}g_{T}h_{T}\right)}{b_{S}f_{S}^{2}}x_{k}+\frac{g_{T}^{2}}{f_{T}^{2}}\right)\right]\right\}\\ =\frac{1}{\sqrt{2\pi }}E_{x_{k}|Y_{1:k}}\left\{\exp\left[-\frac{1}{2}\left({\left(R_{1T}x_{k}-R_{2T}\right)}^{2}-R_{3T}\right)\right]\right\}\\ =\frac{1}{\sqrt{2\pi }}\exp\left[-\frac{1}{2}R_{3T}\right]E_{x_{k}|Y_{1:k}}\left[\exp\left(-\frac{1}{2}{\left(R_{1T}x_{k}-R_{2T}\right)}^{2}\right)\right] \end{array}$$

where

$$R_{1T}=\sqrt{\frac{D_{1T}f_{S}^{2}+b_{S}h_{T}^{2}}{b_{S}f_{S}^{2}}},R_{2T}=\frac{D_{2T}f_{S}^{2}-b_{S}g_{T}h_{T}}{\sqrt{b_{S}f_{S}^{2}\left(D_{1T}f_{S}^{2}+b_{S}h_{T}^{2}\right)}},R_{3T}=\frac{g_{T}^{2}}{f_{S}^{2}}-\frac{{\left(D_{2T}f_{S}^{2}-b_{S}g_{T}h_{T}\right)}^{2}}{b_{S}f_{S}^{2}\left(D_{1T}f_{S}^{2}+b_{S}h_{T}^{2}\right)}$$

(I) Based on $x_{k}\sim N({\hat{x}}_{k|k},P_{k|k})$ and Lemma A1, $E_{x_{k}|Y_{1:k}}\left[T_{b}\right]$ could be obtained as follows,

(A34) $$E_{x_{k}|Y_{1:k}}\left[T_{b}\right]=\frac{1}{\sqrt{2\pi }}\exp\left[-\frac{1}{2}R_{3T}\right]\times \sqrt{\frac{1}{R_{1T}^{2}P_{k|k}+1}}\exp\left[-\frac{{\left(R_{2T}-R_{1T}{\hat{x}}_{k|k}\right)}^{2}}{2\left(R_{1T}^{2}P_{k|k}+1\right)}\right]$$

(J) Finally, bringing the final results of $E_{x_{k}|Y_{1:k}}\left[T_{a}\right]$ and $E_{x_{k}|Y_{1:k}}\left[T_{b}\right]$ into Equation (A29), the final results of $E_{x_{k}|Y_{1:k}}\left[T\right]$, i.e., the formula $T_{new}$ in Theorem 2, could be obtained. Due to space limitations, the result of $T_{new}$ is omitted here. Case 2: If $\tau <t_{k}$, (A) When $\tau <t_{k}$, the degradation process is equivalent to a single-phase nonlinear process, and the randomness of the degradation state at the changing point can be omitted. Therefore, based on $\lambda_{2}\sim N(\lambda_{2p},\sigma_{2p}^{2})$ and the $f_{T}(t|\lambda_{2},x_{\tau })$ in Equation (6), the PDF of the lifetime T could be obtained through Lemma A1 as follows,

(A35) $$\begin{array}{l} f_{T}(t)=\int_{-\infty }^{+\infty }f_{T}(t|\lambda_{2},x_{\tau })p(\lambda_{2})d\lambda_{2}\\ ≅\int_{-\infty }^{+\infty }\frac{w-x_{\tau }-\lambda_{2}\left(\int_{\tau }^{t}\mu_{2}(\rho -\tau ;\vartheta_{2})d\rho -(t-\tau )\mu_{2}(t-\tau ;\vartheta_{2})\right)}{\sqrt{2\pi \sigma_{2}^{2}{\left(t-\tau \right)}^{3}}}\exp\left[-\frac{{\left(w-x_{\tau }-\lambda_{2}\int_{\tau }^{t}\mu_{2}(\rho -\tau ;\vartheta_{2})d\rho \right)}^{2}}{2\sigma_{2}^{2}(t-\tau )}\right]p(\lambda_{2})d\lambda_{2}\\ ≅\frac{1}{\sqrt{2\pi {\left(t-\tau \right)}^{2}\left[{\left(\int_{\tau }^{t}\mu_{2}(\rho -\tau ;\vartheta_{2})d\rho \right)}^{2}\sigma_{2p}^{2}+\sigma_{2}^{2}(t-\tau )\right]}}\times \left[w-x_{\tau }-\left(\int_{\tau }^{t}\mu_{2}(\rho -\tau ;\vartheta_{2})d\rho -(t-\tau )\mu_{2}(t-\tau ;\vartheta_{2})\right)\right.\\ \left.\times \frac{\left(w-x_{\tau })\sigma_{2p}^{2}\int_{\tau }^{t}\mu_{2}(\rho -\tau ;\vartheta_{2})d\rho +\lambda_{2p}\sigma_{2}^{2}(t-\tau \right)}{{\left(\int_{\tau }^{t}\mu_{2}(\rho -\tau ;\vartheta_{2})d\rho \right)}^{2}\sigma_{2p}^{2}+\sigma_{2}^{2}(t-\tau )}\right]\times \exp\left[-\frac{{\left(w-x_{\tau }-\lambda_{2p}\int_{\tau }^{t}\mu_{2}(\rho -\tau ;\vartheta_{2})d\rho \right)}^{2}}{2\left({\left(\int_{\tau }^{t}\mu_{2}(\rho -\tau ;\vartheta_{2})d\rho \right)}^{2}\sigma_{2p}^{2}+\sigma_{2}^{2}(t-\tau )\right)}\right] \end{array}$$

(B) Then based on the relationship between lifetime and RUL, we can further obtain the PDF of RUL considering the unit-to-unit variability as,

(A36) $$\begin{array}{l} f_{L}(l_{k})≅\frac{\left[w-x_{k}-\left(\int_{t_{k}}^{t_{k}+l_{k}}\mu_{2}(\rho -\tau ;\vartheta_{2})d\rho -l_{k}\mu_{2}(t_{k}+l_{k}-\tau ;\vartheta_{2})\right)\times \frac{(w-x_{k})\sigma_{2p}^{2}\int_{t_{k}}^{t_{k}+l_{k}}\mu_{2}(\rho -\tau ;\vartheta_{2})d\rho +\lambda_{2p}\sigma_{2}^{2}l_{k}}{{\left(\int_{t_{k}}^{t_{k}+l_{k}}\mu_{2}(\rho -\tau ;\vartheta_{2})d\rho \right)}^{2}\sigma_{2p}^{2}+\sigma_{2}^{2}l_{k}}\right]}{\sqrt{2\pi l_{k}{}^{2}\left[{\left(\int_{t_{k}}^{t_{k}+l_{k}}\mu_{2}(\rho -\tau ;\vartheta_{2})d\rho \right)}^{2}\sigma_{2p}^{2}+\sigma_{2}^{2}l_{k}\right]}}\\ \times \exp\left[-\frac{{\left(w-x_{k}-\lambda_{2p}\int_{t_{k}}^{t_{k}+l_{k}}\mu_{2}(\rho -\tau ;\vartheta_{2})d\rho \right)}^{2}}{2\left({\left(\int_{t_{k}}^{t_{k}+l_{k}}\mu_{2}(\rho -\tau ;\vartheta_{2})d\rho \right)}^{2}\sigma_{2p}^{2}+\sigma_{2}^{2}l_{k}\right)}\right] \end{array}$$

(C) When considering the three-source variability simultaneously, it is necessary to further incorporate the measurement variability into the PDF of RUL that considers unit-to-unit variability, which is presented as,

(A37) $$\begin{array}{l} f_{L_{k}|Y_{\tilde{\tau }:k}}(l_{k}|Y_{\tilde{\tau }:k})\\ =E_{x_{k}|Y_{\tilde{\tau }:k}}\left\{\frac{1}{\sqrt{2\pi l_{k}{}^{2}\left({\left(\int_{t_{k}}^{t_{k}+l_{k}}\mu_{2}(\rho -\tau ;\vartheta_{2})d\rho \right)}^{2}\sigma_{2p}^{2}+\sigma_{2}^{2}l_{k}\right)}}\right.\times \left[w-x_{k}-\left(\int_{t_{k}}^{t_{k}+l_{k}}\mu_{2}(\rho -\tau ;\vartheta_{2})d\rho -l_{k}\mu_{2}(t_{k}+l_{k}-\tau ;\vartheta_{2})\right)\right.\\ \left.\left.\times \frac{(w-x_{k})\sigma_{2p}^{2}\int_{t_{k}}^{t_{k}+l_{k}}\mu_{2}(\rho -\tau ;\vartheta_{2})d\rho +\lambda_{2p}\sigma_{2}^{2}l_{k}}{{\left(\int_{t_{k}}^{t_{k}+l_{k}}\mu_{2}(\rho -\tau ;\vartheta_{2})d\rho \right)}^{2}\sigma_{2p}^{2}+\sigma_{2}^{2}l_{k}}\right]\times \exp\left[-\frac{{\left(w-x_{k}-\lambda_{2p}\int_{t_{k}}^{t_{k}+l_{k}}\mu_{2}(\rho -\tau ;\vartheta_{2})d\rho \right)}^{2}}{2\left({\left(\int_{t_{k}}^{t_{k}+l_{k}}\mu_{2}(\rho -\tau ;\vartheta_{2})d\rho \right)}^{2}\sigma_{2p}^{2}+\sigma_{2}^{2}l_{k}\right)}\right]\right\} \end{array}$$

where $\tilde{\tau }$ denote the index of the changing point location. (D) Then, based on $x_{k}\sim N({\hat{x}}_{k|k},P_{k|k})$ and Lemma A1, we could obtain the PDF of RUL with three-source variability simultaneously when $\tau <t_{k}$ as follows,

(A38) $$\begin{array}{l} f_{L_{k}|Y_{\tilde{\tau }:k}}(l_{k}|Y_{\tilde{\tau }:k})=\frac{M_{2}-N_{2}\times \frac{\left(w-\lambda_{2p}\int_{t_{k}}^{t_{k}+l_{k}}\mu_{2}(\rho -\tau ;\vartheta_{2})d\rho \right)P_{k|k}+\upsilon_{2}(l_{k}){\hat{x}}_{k|k}}{P_{k|k}+\upsilon_{2}(l_{k})}}{\sqrt{2\pi l_{k}{}^{2}\upsilon_{2}^{2}(l_{k})\left(P_{k|k}+\upsilon_{2}(l_{k})\right)}}\\ \times \exp\left[-\frac{{\left(w-{\hat{x}}_{k|k}-\lambda_{2p}\int_{t_{k}}^{t_{k}+l_{k}}\mu_{2}(\rho -\tau ;\vartheta_{2})d\rho \right)}^{2}}{2\left(P_{k|k}+\upsilon_{2}(l_{k})\right)}\right] \end{array}$$

where

$$\begin{array}{l} \upsilon_{2}(l_{k})={\left(\int_{t_{k}}^{t_{k}+l_{k}}\mu_{2}(\rho -\tau ;\vartheta_{2})d\rho \right)}^{2}\sigma_{2p}^{2}+\sigma_{2}^{2}l_{k},\\ M_{2}=w\upsilon_{2}(l_{k})-\left(w\sigma_{2p}^{2}\int_{t_{k}}^{t_{k}+l_{k}}\mu_{2}(\rho -\tau ;\vartheta_{2})d\rho +\lambda_{2p}\sigma_{2}^{2}l_{k}\right)\left(\int_{t_{k}}^{t_{k}+l_{k}}\mu_{2}(\rho -\tau ;\vartheta_{2})d\rho -l_{k}\mu_{2}(t_{k}+l_{k}-\tau ;\vartheta_{2})\right)\\ N_{2}=\upsilon_{2}(l_{k})-\sigma_{2p}^{2}\int_{t_{k}}^{t_{k}+l_{k}}\mu_{2}(\rho -\tau ;\vartheta_{2})d\rho \left(\int_{t_{k}}^{t_{k}+l_{k}}\mu_{2}(\rho -\tau ;\vartheta_{2})d\rho -l_{k}\mu_{2}(t_{k}+l_{k}-\tau ;\vartheta_{2})\right) \end{array}$$

In this way, the proof has been completed. □
